# Photochemical Impacts from Resolving Isomers and Interference Biases in PTR-ToF-MS Measurements of Atmospheric VOCs

**DOI:** 10.1021/acs.est.6c08699

**Published:** 2026-08-05

**Authors:** Xin Feng, Lirong Hui, Yi Chen, Penggang Zheng, Megan S. Claflin, Brian M. Lerner, Yao Chen, Jiali Zhong, Dasa Gu, Yang Xu, Yijie Yao, Mengyuan Chu, Jia Guo, Qi Ying, Jian Zhen Yu, Douglas Worsnop, Zhe Wang

**Affiliations:** † Division of Environment and Sustainability, 58207 The Hong Kong University of Science and Technology, Kowloon 999077, Hong Kong SAR, China; ‡ Department of Chemistry, The Hong Kong University of Science and Technology, Kowloon 999077, Hong Kong SAR, China; § School of the Environment and Sustainable Engineering, Eastern Institute of Technology, Ningbo 315200, China; ∥ Aerodyne Research, Inc., Billerica, Massachusetts 01821, United States; ⊥ Environmental Central Facility, The Hong Kong University of Science and Technology, Kowloon, 999077, Hong Kong SAR, China

**Keywords:** volatile organic compounds (VOCs), PTR-ToF-MS, gas chromatography (GC), photochemical pollution

## Abstract

While proton-transfer-reaction time-of-flight mass spectrometry (PTR-ToF-MS) is widely used for ambient volatile organic compounds (VOCs) quantification, its accuracy is limited by isomeric speciation and ionization byproducts. Here, gas chromatography (GC) coupled with PTR-ToF-MS was deployed at a suburban site in Hong Kong to resolve isomers and quantify interferences for ambient VOCs measurement. We identified 48 compounds using GC-PTR and resolved their isomer profiles in direct-PTR data. Our analysis revealed that direct-PTR measurements substantially underestimated long-chain aldehydes (C_5_–C_8_) due to extensive fragmentation, while overestimating isoprene, benzene, styrene, and phenol by ∼14–60% because of interference from other species. The biogenic VOCs’ OH reactivity was overestimated by up to 31% for monoterpenes and 241% for isoprene, depending on the seasonality of biogenic emissions. Propagating these biases into the photochemical model resulted in a 46% overestimation in daytime net O_3_ production under VOC-limited and low-isoprene conditions, whereas the overestimation decreased to 19% under VOC-saturated, high-isoprene conditions. Correcting isomer distributions and interference effects reduced modeled ozone production rates and altered precursor sensitivities, revealing a larger role for oxygenated VOCs in ozone formation than previously recognized. Our results highlight the necessity for isomer-resolved measurements and interference-aware calibration to improve VOC-based assessments of photochemical air pollution.

## Graphical abstract

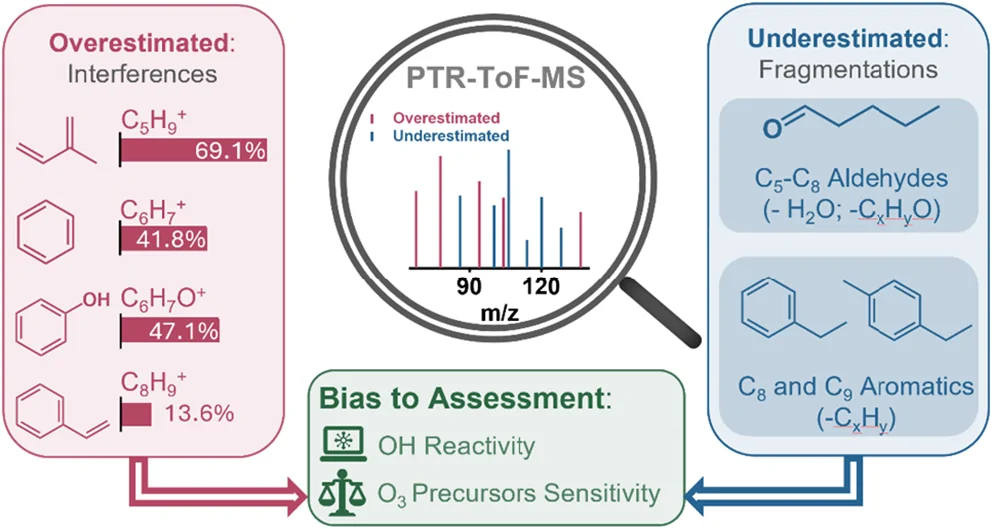

## 1 Introduction

Volatile organic compounds (VOCs) are key precursors of tropospheric ozone (O_3_) and secondary organic aerosols (SOA), influencing both air quality and climate change, and many also pose direct health risks.^1–3^ Various VOCs are present in ambient air at trace levels, and they originate from both anthropogenic sources (*e.g*., fossil fuel combustion, solvent evaporation, cooking activities) and natural processes (*e.g*., biogenic emissions, oceanic emissions).^4^ Among them, oxygenated VOCs (OVOCs), including alcohols, carbonyls, and acids, are particularly reactive due to their functional groups.^5–8^ They arise from both primary emissions and atmospheric oxidation of hydrocarbons.^9,10^ Accurate quantification of VOCs and OVOCs is crucial for identifying their sources and assessing their contributions to O_3_ and SOA formation.

Proton-transfer-reaction time-of-flight mass spectrometry (PTR-ToF-MS) enables rapid and sensitive detection of a wide variety of VOCs, including alkenes, aromatics, OVOCs, and nitrogen-containing compounds.^11,12^ Because of its high sensitivity, broad chemical coverage, and subsecond response time, this technique has been extensively applied to measure reactive, trace-level species across diverse environments,^13–18^ supporting detailed investigations of VOC abundances, emission fluxes, source signatures, and photochemical contributions.^5,19–22^ However, in addition to the proton-transfer products ([MH]^+^) used for quantification, secondary ion–molecule reactions including fragmentation and water clustering can generate additional product ions (*e.g*., [MH-C_*x*_H_*y*_O_*z*_]^+^, [MH - H_2_O]^+^, and [MH+H_2_O]^+^), producing potential measurement uncertainties. Fragmentation is particularly prevalent in species with larger molecular weight (MW) or specific functional groups, such as cycloalkanes, alkyl aromatics, alcohols, and aldehydes.^23–25^ These additional product ions can overlap with lower-mass target quantitative ions ([MH]^+^), leading to their overestimation, and simultaneously alter parent ion calibration factors for noncalibrated species, resulting in underestimation. Together, these effects may introduce substantial bias into PTR-based assessments of VOC concentrations and their atmospheric impacts. Furthermore, PTR-ToF-MS provides only the molecular mass of VOC species, which may be associated with multiple isomers. The inability to resolve isomeric species limits accurate source apportionment and an understanding of their atmospheric transformations.

To address these challenges, several studies have coupled gas chromatography (GC) with PTR-ToF-MS to characterize isomer and product distributions. Warneke et al.^26^ and Koss et al.^27^ used gas standard mixtures and biomass burning samples to map the major contributors to PTR signals. More recently, field-deployable GC-PTR-ToF-MS systems have advanced this approach, quantifying interference ions and product ion distributions in indoor and urban air.^24,28–30^ In cities such as Los Angeles and Shanghai, interference ions have been shown to contribute more than 40% of signals typically attributed to compounds like isoprene (C_5_H_9_^+^) and benzene (C_6_H_7_^+^).^24,30^ The same systems have also been used to characterize the isomer profiles for key VOC species, including monoterpenes and C_8_- and C_9_-aromatics in urban and rural environments.^16,17,29,30^ However, the isomer distribution and interference-ion contributions vary significantly across different locations and seasons. Instrument settings, including reactor reduced electric field strength (E/N), ion optic voltage gradients, and quadrupole settings, further modify product ion distributions, thereby affecting the presence of interference ions.^25,31^ Approaches employed in previous studies to characterize these effects and mitigate such interferences were largely instrument- and campaign-dependent. Moreover, previous attempts at resolving isomer profiles have mainly focused on aromatics and monoterpenes, but relatively little attention has been paid to OVOC isomers, particularly those with higher carbon numbers. The uncertainties and biases introduced by unresolved isomers and interference ions remain a significant challenge for accurate concentration determination and subsequent photochemical process assessment.

In this study, we conducted field measurements at a suburban coastal site using a GC-PTR-ToF-MS system to resolve isomeric contributions and quantify product ion distributions. We used isomer fractions derived from GC analyses to separate isomers in direct-PTR measurements and to improve the quantification of PTR-detected species. We further identified interference ions affecting isoprene, benzene, phenol, and styrene and corrected their real-time signals. Finally, we assessed how uncertainties in isomer distributions and interference-ion corrections influence OH reactivity estimates and the quantification of key photochemical processes.

## 2 Materials and Methods

### 2.1 Site Description

Field measurements were conducted at a suburban coastal site in eastern Hong Kong from 15 November to 29 December, 2022, covering partial autumn and winter. The measurement site is located at the Hong Kong University of Science and Technology Air Quality Research Supersite (22.33°N, 114.27°E, 154 m above sea level), which is approximately 5–10 km away from the urban region. The site is less affected by urban anthropogenic activities except for a nearby dormitory construction project, a few campus vehicle traffic, and household activities. The site often receives transported pollution from urban Hong Kong and the Pearl River Delta (PRD) region, as well as long-range air masses from eastern and southern China driven by the Asian monsoon.^5,32^ Continuous measurements of trace gases (such as O_3_, NO, NO_2_, HONO) and meteorological parameters (temperature, relative humidity, winds, and irradiance) were recorded throughout the field campaign.

### 2.2 VOCs Measurement by Direct-PTR and GC-PTR

A Vocus 2R PTR-ToF-MS (Aerodyne Research, Inc. and Tofwerk AG)^12^ was deployed for continuous, real-time VOC measurements. Ambient air was drawn through a 3.5 m, 1/4″ O.D., inert silicon-coated stainless-steel inlet at 15 L/min (LPM). The Vocus PTR subsampled 3 LPM from the manifold via a 2.5 m, 1/4″ I.D. perfluoroalkoxy alkane (PFA) tube heated to 40 °C. 80 sccm of that flow entered the Vocus capillary inlet, and the remainder was exhausted. The focusing ion-molecule reactor (FIMR) was operated at 2.5 mbar and 100 °C with front and back voltages of 550 and 46 V, resulting in an E/N ratio of approximately 100 Td. The skimmer and BSQ voltages were set at 0.88 V and −2.53 V, respectively. Mass spectra were collected from *m*/*z* 30 to 400 with a mass resolution of ∼10,000 *m/*Δ*m* for C_8_H_10_H^+^ (*m*/*z*, 107). Instrument background was measured for 1 min each hour using a catalyst-based zero air generator (heated to 400 °C). Peak identification and integration of the mass spectrum were performed with Tofware v3.2.5. Many hundreds of ion peaks can be identified from the Vocus PTR spectrum, but here we only focus on the signals that were resolved and quantified from the coupled GC measurements.

An *in situ* GC equipped with an integrated thermal desorption preconcentration system (Aerodyne Research, Inc.) was coupled to the Vocus PTR to collect and preseparate the isomers before detection by Vocus PTR. Ambient air for the GC sample was collected from the sampling manifold at 100 sccm for 10 min. The sampled gases passed through a sodium sulfite (Na_2_SO_3_) oxidant trap heated to 35 °C to remove O_3_ and then collected on a multibed sorbent tube (Tenax TA/Graphitized Carbon/Carboxen 1000, Markes International), and were subsequently focused onto a multibed focusing trap (Tenax TA/Carbopack X/Carboxen 1003, Markes International). After sampling, the flow was thermally desorbed and injected onto two GC channels (Channel 1: MXT-624, Restek and Channel 2: MXT-WAX, Restek) with a programmed temperature ramp from 35 to 225 °C, producing a 9 min chromatogram (temperature profile provided in Figure S1) for each channel. Further details on this system are available in previous studies.^25,30^ During the field campaign, the system was switched between continuous direct Vocus PTR sampling (direct-PTR, 1 Hz) and GC-PTR sampling. After 3–5 h of direct-PTR measurement, two ambient and one zero samples were collected by GC-PTR to capture the isomer-species profiles.

### 2.3 GC-PTR Peak Identification

The GC retention times (RTs) of species in gas standards were determined during GC-PTR calibration using gravimetrically prepared gas standards containing 15 representative VOCs (Apel-Riemer Environmental, Inc.). The RTs for other nonstandard compounds were estimated based on their retention indices (RIs) from the NIST database and the linear relationships between the known RTs and their RIs.^33^ Chromatographic peak identification and fitting were performed using TERN v2.2.20 (Aerodyne Research, Inc.), after high-resolution peak integration of the PTR mass spectrum in Tofware v3.2.5. It should be noted that pentanal peak fitting required special handling because the pentanal peak in the C_5_H_11_O^+^ chromatogram was partially obscured by a closely eluting 3-pentanone peak and by significant pentanal fragmentation in PTR. Therefore, pentanal quantification was obtained indirectly via fragmentation-correction calculations using its corresponding fragment peak at C_5_H_9_^+^ rather than by direct integration of the C_5_H_11_O^+^ peak. A detailed description of the peak identification and the fragmentation-correction procedure is provided in Text S1.

### 2.4 Calibration Factor Determination and Correction

Calibration factors for PTR measurements were obtained through direct calibration with reference standards or through theoretical estimation based on proton-transfer reaction rate coefficients (*k*_PTR_).^23,34^ For direct-PTR measurements, multipoint calibrations were performed periodically using the same gas standards employed for GC-PTR calibration. Postcampaign calibrations were also conducted to determine calibration factors for additional species, including acetaldehyde, acrolein, methyl isobutyl ketone (MIK), toluene, and styrene. Detailed calibration factors for all calibrated species are given in Table S3.

For compounds not included in the standards, calibration factor estimation is complicated by the formation of additional product ions in PTR-ToF-MS. These ions can distort the linear regression between measured sensitivities and d*k*_PTR_, leading to a systematic underestimation of calibration factors for uncalibrated compounds when using experimental sensitivities. Conversely, uncalibrated compounds may form additional ions themselves, causing an overestimation of their calibration factors. Together, these effects introduce considerable uncertainty into the calibration factors and concentration estimates for uncalibrated compounds. To address this issue, we applied a correction factor (***f***) for each species to account for the fraction of the quantitative ion relative to the total ion signal (quantitative plus additional ions). This factor was used to adjust calibration factors for all uncalibrated VOC species measured by PTR. Further details of the procedure and formula are provided in Text S2, and the resulting ***f*** values and corrected calibration factors are summarized in Table S4.

### 2.5 Photochemical Box Model

The Framework for 0-D Atmospheric Modeling (F0AM, v4.3.0.1) coupled with the Master Chemical Mechanism (MCM v3.3.1) was used to simulate the *in situ* photochemical process of measured VOCs and to assess their impacts on radical budgets and ozone formation.^35^ The model was constrained using the diurnal profile of observed air pollutants and meteorological data (relative humidity, temperature, and sea level pressure) over the period of November 15 to December 19 at a time resolution of 10 min. During this period, all concurrent measurements were available. Higher time-resolution data, including VOCs measured by PTR-ToF-MS (Table S5), NO, NO_2_, O_3_, CO, HONO, and meteorological parameters, were all averaged to 10 min intervals. Detailed information about the instruments can be found in previous studies.^5,8,36^ The photolysis frequencies during the simulation were calculated as a function of solar zenith angle with a correction factor of 0.9 based on concurrent measurement of solar radiation data accounting for cloud and aerosol shading.^35^ Canister nonmethane hydrocarbons (NMHCs) (Table S5) measured by GC-MS/FID/ECD were interpolated using their linear relationships with selected hydrocarbons measured by PTR (*i.e*., C_4_H_8_, C_5_H_10_, C_6_H_10_, C_10_H_14_) and CO.^37^

To assess how uncertainties in isomer assignments and interference ions in PTR-ToF-MS measurements propagate into photochemical simulations, we designed several modeling scenarios. Base Scenario 1 (BS1): VOC inputs were derived from the direct-PTR measurement, and calibration followed common practice from earlier studies, without applying the ion-fraction correction factor ***f***. No interference correction was applied for isoprene, benzene, phenol, and styrene.^5,22,38^ Isomer distributions for aromatics and monoterpenes were obtained from the canister data, while most OVOCs were equally assigned among plausible isomers, except C_3_H_6_O and C_4_H_8_O, which were assigned solely to acetone and methyl ethyl ketone (MEK) following previous treatment for photochemistry investigation using PTR data.^8,21,22,38^ Base Scenario 2 (BS2): VOC inputs were taken directly from the GC-PTR data set, which already incorporates interference corrections and GC-derived isomer concentrations with the ***f-*** value correction incorporated. Figure S7 presents the diurnal profiles of the sum VOCs, O_3_, CO, NO, NO_2_, and HONO mixing ratios constrained in BS2. To further investigate which part of the correction procedure has the greatest impact on photochemical simulation, three sensitivity scenarios were performed by adjusting selected VOC concentrations from BS1. Sensitivity Scenario 1 (SS1) applied GC-PTR-resolved isomer concentrations, including C_8_ and C_9_ aromatics, monoterpenes, and several C_4_–C_8_ carbonyls; Sensitivity Scenario 2 (SS2) corrected concentrations for aromatics (styrene, benzene, and phenol); Sensitivity Scenario 3 (SS3) corrected isoprene concentration. For each scenario, the daytime-averaged net O_3_, RO_2_, and HO_2_ production rates (07:00–19:00 LST) were calculated to assess the deviations in photochemical simulation. The relative incremental reactivity (RIR)^39^ was also calculated to investigate the sensitivity of O_3_ production rate to the VOC precursors in GC-PTR and direct-PTR data sets. The RIR is defined as the ratio of the decrease in net O_3_ production in daytime to the reduction in the concentration of a given O_3_ precursor (eq S5). A 10% reduction in precursor concentration was applied in this study. Additional details on the model description are provided in Text S3.

## 3 Results and Discussion

### 3.1 Overall Results and Comparison between Direct-PTR and GC-PTR Measurements

Following the isomer identification approach, we identified and quantified 48 compounds in the GC-PTR chromatograms from channel 1, including 7 alkenes, 13 aromatics, 14 aldehydes, and 14 ketones (Table S2). In direct-PTR measurements, these compounds are represented by 17 quantitative ion signals. Among them, 9 signals were primarily attributed to groups of VOC isomers with different structures and reactivities, including monoterpenes (C_10_H_16_), C_8_ and C_9_ aromatics (C_8_H_10_ and C_9_H_12_), and C_4_–C_8_ carbonyls (C_4_H_6_O, C_4_H_8_O, C_5_H_10_O, C_6_H_12_O, C_7_H_14_O, and C_8_H_16_O). The remaining 8 signals were dominated either by a single VOC compound or a mixture of one compound and interference ions from other species. Accurate quantification of these isomer groups using PTR-ToF-MS is inherently challenging because calibration factors vary across isomers due to differences in the *k*_PTR_ and ion-fragment distributions. Furthermore, additional product ions from certain compounds within isomer groups can interfere with the quantification of the other key species. Therefore, distinguishing individual isomer signals within direct-PTR measurements is critical for reliable VOC quantification.

To achieve this, we combined direct-PTR measurements with temporally adjacent GC-PTR samples to separate isomer contributions for the corresponding molecular formulas. As an example, the C_4_H_7_O^+^ signal was resolved into contributions from methacrolein (MACR), methyl vinyl ketone (MVK), and 2-butanal, using their fractional abundances derived from the GC-Vocus ambient sample (Figure S8). These fractions were then applied to the direct-PTR signal to reconstruct time series for individual isomers (Figure 1). Concentrations were calculated using isomer-specific calibration factors derived from the GC-PTR data set as described in Section 2.4 (Tables S3 and S4). To assess biases associated with conventional PTR-ToF-MS approaches, we also calculated concentrations using calibration methods commonly applied in previous studies that do not account for ion-fraction corrections,^5,22,38^ hereafter referred to as the direct-PTR data set.

**Figure 1:**
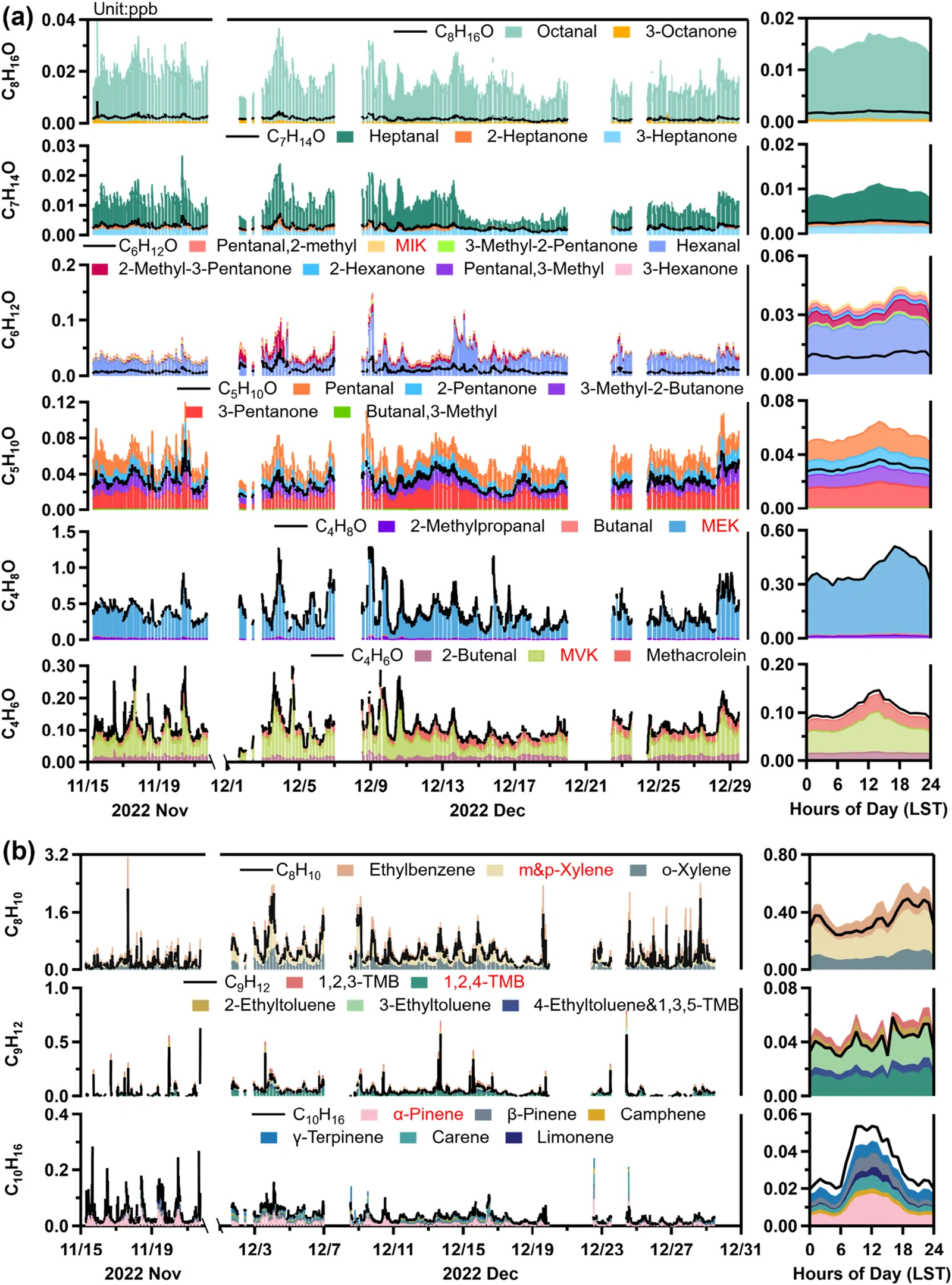
Time series (left) and diurnal variation (right) of OVOCs (a) and VOCs (b) mixing ratios in GC-PTR data set measurements during the campaign, with the direct-PTR data set mixing ratios overlaid. Red-labeled species in the legend were used for direct-PTR calibration.

Figure 1 compares mixing ratios derived from direct-PTR and GC-PTR data sets across different isomer groups. For most calibrated compounds (marked red in legends), direct-PTR concentrations agreed with GC-PTR values within a factor of 0.8–1.1, except for C_6_H_12_O. Agreement was strongest for compound groups dominated by isomers with similar reaction rates and limited fragmentation, indicating that PTR quantification performs well under these conditions. Specifically, for C_4_H_6_O carbonyls (as C_4_H_7_O^+^ signals), MACR, 2-butanal, and MVK were the dominant contributors. Their similar k_PTR_ values and minimal fragmentation (*f* > 0.92; Table S4) resulted in comparable calibration factors, leading to close agreement between direct-PTR and GC-PTR concentrations. For C_4_H_8_O compounds, MEK dominated the C_4_H_9_O^+^ signal and was used as calibration standard during the campaign. As a result, direct-PTR concentrations for C_4_H_6_O and C_4_H_8_O closely align with the summed concentrations of their isomers from the GC-PTR data set, with average ratios of 1.1 and 1.0, respectively.

In contrast, monoterpene concentrations in the direct-PTR data set were overestimated by about 14%, primarily due to calibration based on α-pinene only. In PTR, many monoterpene isomers fragment strongly to C_6_H_9_^+^,^40^ and α-pinene has a lower *f* value (0.45) and calibration factor than camphene (*f*: 0.48), γ-terpinene (*f*: 0.48), and limonene (*f*: 0.63), which was consistent with the fragmentation pattern observed by previous studies.^28,41^ Applying the α-pinene calibration factor to these isomers therefore inflates total monoterpene concentrations. Direct-PTR concentrations for C_8_ and C_9_ aromatics were underestimated by approximately 18%. Although calibrated using m-xylene and 1,2,4-trimethylbenzene (TMB), other important isomers, such as ethylbenzene and 2- and 3-ethyltoluene, fragment more extensively into C_6_H_7_^+^ and C_7_H_9_^+^, yielding lower quantitative-ion fractions (***f*** values) and thus lower effective sensitivities. Meanwhile, fragmentation ions lead to significant overestimation of benzene.^24^ ,^25^ ,^30^ As shown in Figures S9a and S10a, benzene accounted for only 60% of total peak areas in the C_6_H_7_^+^ chromatogram, while fragmentation of ethylbenzene and benzaldehyde contributed 29% and 11%, respectively, similar to previous observations in the urban region.^24,42^ Signals commonly assigned to styrene (C_8_H_9_^+^) and phenol (C_6_H_7_O^+^) were also affected by fragmentation from other aromatic compounds. Although styrene dominated the C_8_H_9_^+^ signal, approximately 18% on average originated from *m*- and *p*-xylene (Figures S9b and S10b). Phenol could not be separated through the MXT-624 column; however, the C_6_H_7_O^+^ chromatograms revealed multiple interference peaks associated with benzene, toluene, ethylbenzene, and benzaldehyde (Figure S9c). Although the exact formation mechanism of C_6_H_7_O^+^ could not be resolved in this study, it may arise from secondary ion–molecule reactions and/or fragmentation processes within the PTR reactor. Previous work has suggested that this ion is unlikely to be generated through O_2_^+^ or NO^+^ chemistry under typical PTR-MS operating conditions.^25^ Further experimental and theoretical investigations are needed to elucidate its formation mechanism.

In contrast, C_5_–C_8_ carbonyl concentrations were substantially underestimated in direct-PTR measurements, by factors of 1.8 to 8.1, due to extensive fragmentation of long-chain aldehydes. For example, although MIK was used to calibrate the C_6_H_13_O^+^ signal, GC-PTR-derived C_6_ carbonyl concentrations were still 3.8 times higher than that from direct-PTR. As explained in Text S1 and Figure S4, 91% of the pentanal was fragmented to C_5_H_9_^+^ signals, while the direct-PTR signal of C_5_H_11_O^+^ was mainly attributed to 2-pentanone, 3-pentanone, and 3-methyl-2-butanone. Similarly, hexanal and heptanal contributed minimally to their parent ions (C_6_H_13_O^+^ and C_7_H_15_O^+^) but fragmented extensively into dehydrated ions (*i.e*., C_6_H_11_^+^ and C_7_H_13_^+^), as shown in the GC chromatograms in Figure S11a,b. Although octanal was the dominant contributor to the C_8_H_17_O^+^ signal (Figure S11c), it still fragmented substantially to the C_8_H_15_^+^ and C_5_H_9_^+^ signals. These unaccounted fragmentation pathways of long-chain aldehydes lead to substantial underestimation of carbonyl concentrations but overestimation of other VOCs, especially isoprene, in conventional PTR measurements.

As shown by the ambient GC-PTR chromatograms of the C_5_H_9_^+^ signal in Figure S9d,e, fragmentation of long-chain aldehydes, particularly pentanal, octanal, and nonanal, was the dominant source of interference, accounting for an average of 81.5% of the total peak areas in the chromatogram of ambient samples (Figure S10b). Comparable levels of interference have been reported in forested, coastal, and urban environments, reflecting emissions from biogenic sources, ocean surfaces, cooking activities, and secondary formation.^16,18,24,30^ Additionally, two unidentified C_9_H_17_^+^ peaks (RTs: 320.7 s and 327.1 s) contributed an additional 4.3% on average in ambient C_5_H_9_^+^ chromatograms.

### 3.2 Resolved Isomer Profiles and Diurnal Variation

The fractional contributions of the isomers within each group are presented in Figure 2. MACR (57.6%) and MVK (27.0%) were identified as the predominant contributors to C_4_H_6_O carbonyl concentrations and showed strong midday enhancements (Figure S12a), consistent with secondary formation from isoprene oxidation.^38^ 2-Butenal was also an important C_4_H_6_O isomer but exhibited a relatively stable diurnal profile (Figure 1a), suggesting a stronger influence from continuous anthropogenic sources (*e.g*., biomass burning, volatile chemical products).^24,27^ Within the C_4_H_8_O carbonyl group, MEK accounted for approximately 94.9% of total concentrations (Figures 1a and 2a), with minor contributions from 2-methylpropanal and butanal. MEK showed clear enhancement during early evening (Figure 1a), which reflects a combination of increased traffic-related and solvent emissions,^43^ continued contribution from daytime secondary formation, and reduced vertical mixing during boundary-layer collapse. In contrast, the daytime increase in the 2-methylpropanal fraction suggested more secondary atmospheric formation (Figure S12b).

**Figure 2:**
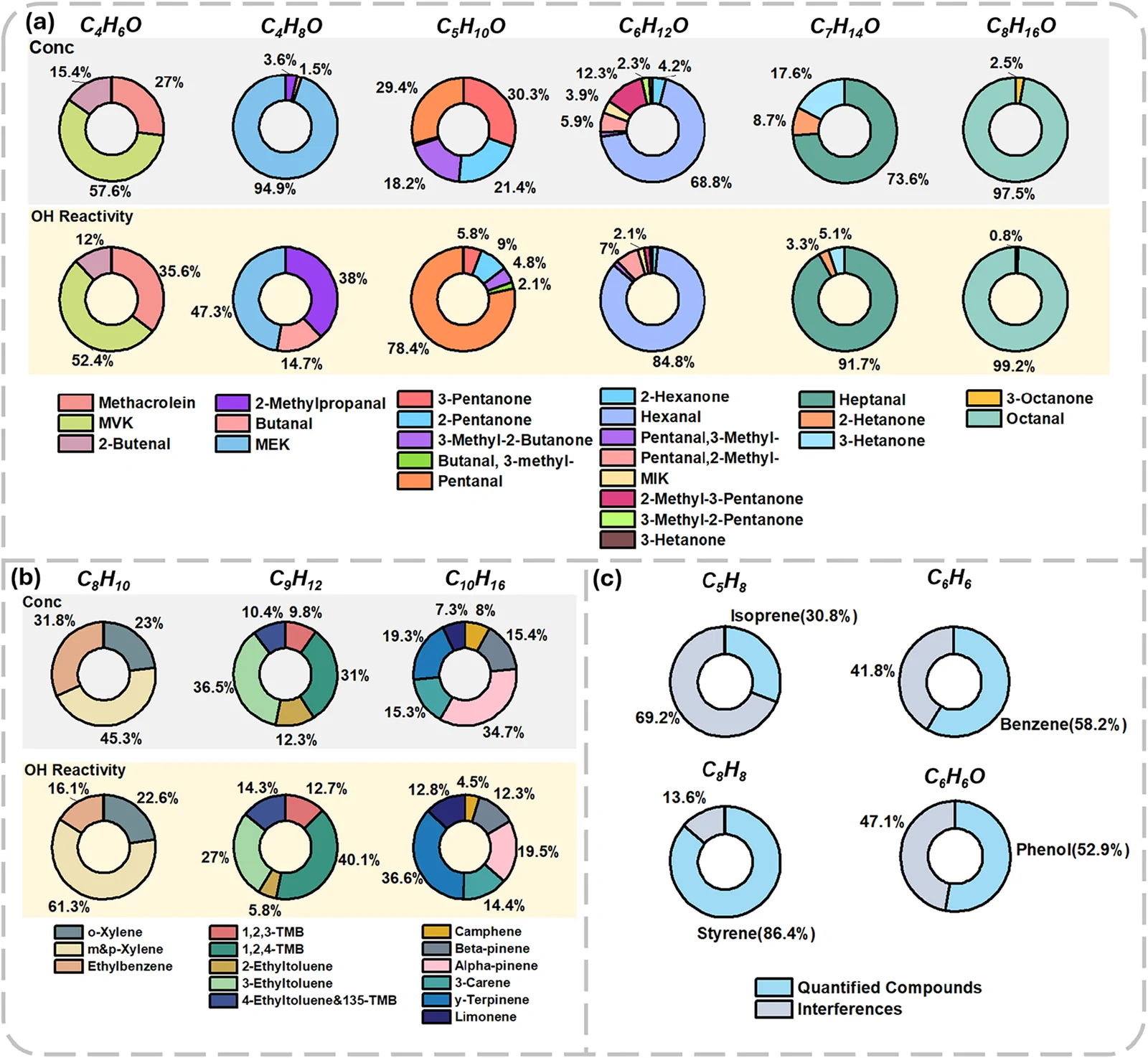
Fractions of the OVOC (a) and VOC (b) isomers concentration and their contribution to the OH reactivity among isomers. (c) Pie chart illustrating the contributions of quantified compounds (isoprene, benzene, styrene, and phenol) and interferences to direct-PTR measurements, as determined by the correction method described in Section 3.3.

The isomer fractions of C_5_–C_8_ carbonyls remained relatively stable throughout the diurnal cycle, as presented in Figure S12c–f. As illustrated in Figure 2a, C_5_ carbonyls (C_5_H_10_O) consisted mainly of 3-pentanone (30.3%), pentanal (29.4%), 2-pentanone (21.4%), and 3-methyl-2-butanone (18.2%). Aldehydes dominated the C_6_, C_7_, and C_8_ carbonyls, with hexanal (68.8%), heptanal (73.6%), and octanal (97.5%) comprising the majority of each respective group (Figure 2a). Most aliphatic carbonyls exhibited peak concentrations between 14:00 and 15:00 LST, indicating strong photochemical production (Figure 1a). In contrast, hexanal reached the maximum concentration in the late afternoon, around 18:00 LST (Figure 1a), likely reflecting a combination of secondary formation and primary emissions from cooking-related activities.^29^

The isomer distribution of two typical aromatic compounds, C_8_H_10_ and C_9_H_12_, was also investigated (Figure 2b). For C_8_ aromatics, m-/*p*-xylene (45.3%) were the most abundant isomers, followed by ethylbenzene (31.8%) and o-xylene (23.0%). For C_9_ aromatics, 3-ethyltoluene (36.5%) and 1,2,4-trimethylbenzene (TMB, 31.0%) were the most abundant isomers. As shown in Figure S12g–h, the fractions of m-/*p*-xylene and 1,2,4-TMB decreased around midday, which is consistent with their higher OH reactivity and more rapid daytime oxidation compared with other isomers. Among monoterpenes (C_10_H_16_), α-pinene (34.7%), γ-terpinene (19.3%), and β-pinene (15.4%) made substantial contributions (Figure 2b). Unlike other monoterpenes that peak around noon due to temperature- or solar-dependent biogenic emissions, camphene and γ-terpinene exhibited relatively flatter diurnal patterns (Figure 1b), suggesting potentially different biogenic mechanisms or other anthropogenic emissions, as well as loss mechanism.^13,14,16^ Additionally, limonene exhibited a more pronounced diurnal variation, with its fractional contribution decreasing to ∼0.02 at night and increasing to ∼0.15 during the day (Figure S12i). This larger fluctuation, relative to α-pinene and β-pinene, is likely associated with enhanced emissions from limonene-rich sources, such as fragranced volatile chemical products associated with routine human activities.^13^

### 3.3 Correction of Interference in the Direct-PTR Data Set

Several methods have been proposed to correct PTR-based isoprene measurements, particularly in forest environments.^16,30^ However, most previous studies considered only aldehydes with more than seven carbon atoms and assumed that aldehydes dominate the isomer mixture. Pentanal fragmentation is typically neglected, which can lead to discrepancies between corrected PTR isoprene concentrations and GC-based measurements.^30^ Coggon et al.^24^ proposed a correction method based on the nighttime ratio between the direct-PTR C_5_H_9_^+^ signal and the sum of C_8_H_17_^+^ and C_9_H_19_^+^ signals (dehydrate signal of octanal and nonanal), assuming that isoprene levels approach zero at night and that C_5_H_9_^+^ signal is primarily produced by dehydration of aldehydes. While effective under certain conditions, this method can underestimate isoprene or even produce negative nighttime values when other nonbiogenic isoprene sources are present, as observed in previous studies.^30^ To address these limitations, we introduced a correction to direct-PTR measurements by explicitly subtracting contributions from interference ions for isoprene and three additional VOCs. Full details of the correction procedure are provided in Text S4.

Figure 3 illustrates the diurnal profiles of these species before and after correction. Corrected isoprene concentrations (Figure 3a) presented much lower values during nighttime (∼0.02 ppb) and decreased by an average of 0.17 ppb during daytime hours (7:00–18:00 LST). Similar to a previous study,^24^ the contribution of interference was predominant in nighttime and wintertime (Figure 3b,c) due to less isoprene emissions from biogenic sources. However, the interference fraction was 69.2% (Figure 2c), which was lower than that directly estimated by the GC-PTR ambient sample. This difference is likely due to internal aldehydes (*e.g*., pentanal) formed by reactions between sorbent tube materials and residual ambient O_3_ in the GC system, leading to an overestimation of aldehyde contributions from the GC-PTR sample chromatogram.^16^ Similarly, the averaged benzene concentrations were also reduced from 0.55 to 0.32 ppb after correction, with interference accounting for 41.8% (Figure 2c). Benzene and ethylbenzene mostly originate from vehicle and fossil fuel combustion in urban areas,^27,44,45^ while benzaldehyde may originate from vehicle emissions, biomass burning, cooking, and secondary production. The interference had previously produced an apparent afternoon benzene peak around 14:00 LST, which disappeared after correction. The averaged concentrations of styrene and phenol (Figure 3e,f) decreased from 0.59 to 0.51 ppb and from 0.17 to 0.09 ppb, corresponding to interference contributions of 13.6% and 47.1%, respectively. Additionally, rush-hour phenol peaks at 8:00 LST and 18:00 LST were largely eliminated, leaving only a modest midday enhancement, indicative of photochemical secondary formation.

**Figure 3:**
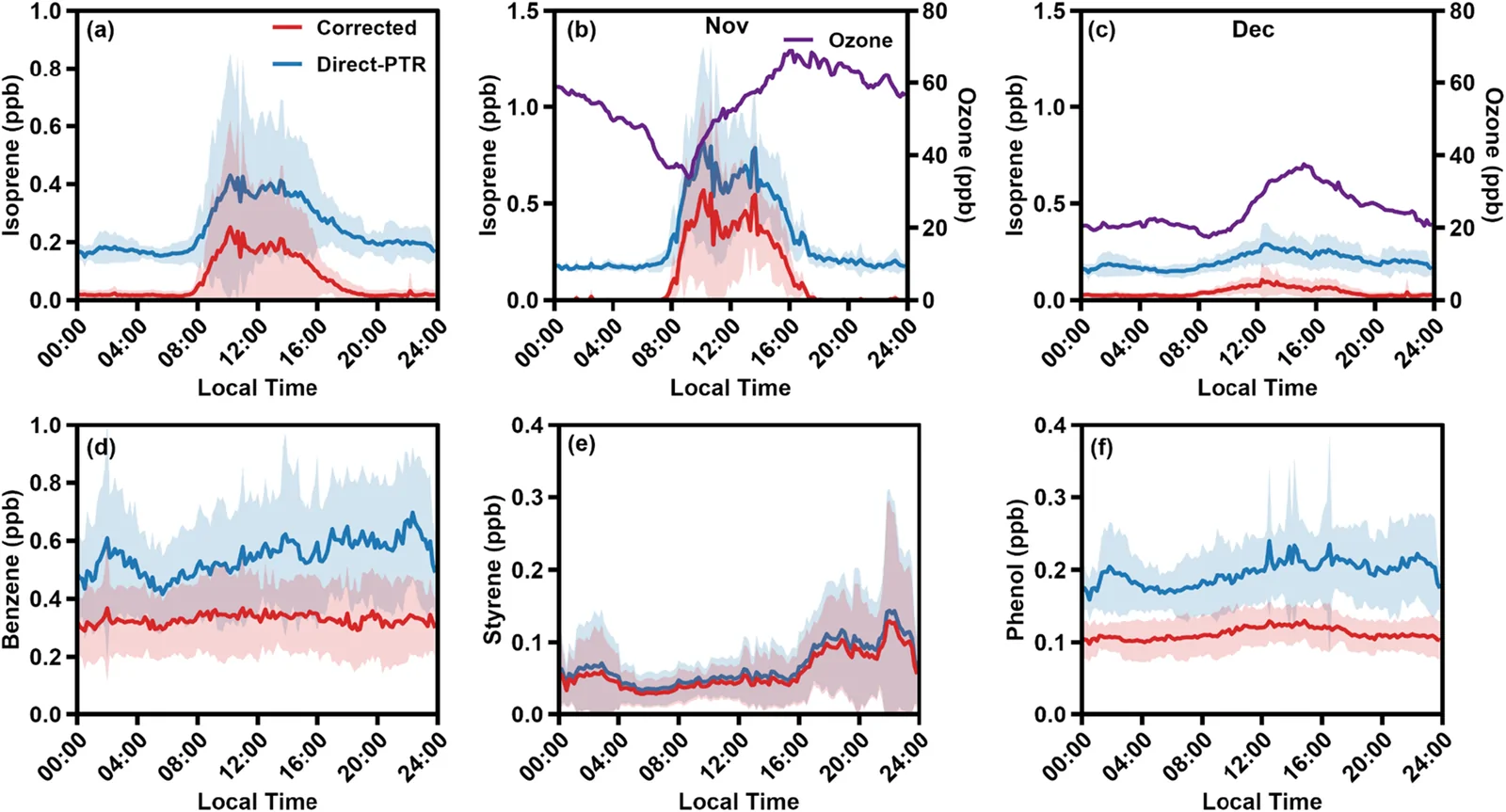
Diurnal variations of isoprene determined by direct-PTR and GC-PTR correction (a), and the diurnal variations of isoprene and O_3_ in November (b) and December (c). (d–f) Diurnal variations of benzene (d), styrene (e), and phenol (f) from direct-PTR and GC-PTR corrected data.

Correspondingly, we compared our corrected isoprene and benzene concentrations with values determined using the method of Coggon et al.^24^ The two approaches agreed well, while the Coggon-based method overestimated the isoprene mixing ratios (Figure S13a,c), likely due to the omission of pentanal from sources that are not correlated with nonanal and octanal (*e.g*., pentanal from vehicle emissions). Building on the relatively stable isomeric composition of C_5_–C_8_ carbonyls observed in Section 3.2, we further propose an alternative correction method when concurrent GC-PTR measurements are not available. Specifically, fragmentation ratios of three aldehydes to C_5_H_9_^+^ (*r*_1_), which are normally constant values although dependent on instrument settings,^25^ could be obtained from calibrations with individual standards or literature values, while aldehyde fraction (*r*_2_) within the total PTR-measured carbonyl signal (MH_PTR_^+^) could be characterized from prior GC-PTR measurements or other offline analytical methods. The corrected isoprene signal can then be obtained from

1 $${{C_{5}H_{9}}^{+}}_{isoprene}={{C_{5}H_{9}}^{+}}_{PTR}-\sum r_{1}\times r_{2}\times {MH^{+}}_{PTR}$$

Here, the *r*_1_ values were 20.8, 4.5, and 2.3 for pentanal, octanal, and nonanal, respectively, and the *r*_2_ values were 0.025 and 0.875, with a variation range of 0.014–0.050 for pentanal (C_5_H_11_O^+^) and 0.679–0.999 for octanal (C_8_H_17_O^+^) over 1.5 months of measurements. The C_9_H_19_O^+^ signal was dominated by nonanal; thus, the *r*_2_ was 1 for nonanal. These results suggest relatively stable ratios that can be used for correction of other periods and conditions. Isoprene concentrations determined using this simplified method exhibited a strong correlation with those obtained from the GC-based correction approach, with a slope of 1.02 and *R* ^2^ of 0.98 (Figure S14). The pentanal-based correction is only applicable under low E/N conditions that allow retrieval of a measurable pentanal signal, as pentanal fragments heavily at higher E/N. Because the exact sources of the unknown C_9_H_17_^+^ signals are not well understood, likely contributed by some cycloalkanes, they were not included in our simplified correction method. Future work is needed to quantify this interference.

For benzene, Coggon et al.^24^ illustrated that the direct-PTR signal of C_6_H_6_^+^, the charge transfer product ion which forms via reaction with O_2_^+^ in the ion-molecule reactor, is largely free of interference and provides a reliable independent estimate of benzene concentration. As shown in Figure S13b,d, benzene mixing ratios derived from direct-PTR signal of C_6_H_6_^+^ aligned well with those obtained using our correction method, confirming the robustness of this alternative approach for PTR-based benzene quantification.

### 3.4 Implications for OH Reactivity Estimates

We further explored how these uncertainties affect estimates of VOC OH reactivities. A detailed description of the estimation method is provided in Text S5. Except for C_4_H_6_O, OH reactivity from OVOCs was significantly underestimated in the direct-PTR data set (Figure 4a), particularly for C_5_–C_8_ carbonyls, with underestimation rates ranging from 57% to 95%. This is primarily due to the higher reactivity of long-chain aldehydes compared with other isomers and their severely underestimated concentrations in direct-PTR measurements. C_4_H_8_O also exhibited a 50% underestimation in OH reactivity, due to the unaccounted contribution from 2-methylpropanal and butanal. These two isomers represented only 3.6% and 1.5% of the measured concentration, respectively, yet contributed 14.7% and 38.0% of the total OH reactivity within the C_4_H_8_O group (Figure 2a). In addition, OH reactivity from C_8_ and C_9_ aromatics was also underestimated by 22.1% and 28.6% in the direct-PTR data set, respectively (Figure 4b). This bias is attributed to the underrepresentation of highly reactive species, such as m-/*p*-xylene and 1,2,4-TMB in conventional PTR measurements (Figure 2b). In contrast, the OH reactivity of monoterpenes was overestimated (∼31%) in the direct-PTR data set due to inflated concentration estimates (Figure 4b). Notably, γ-terpinene contributed 36.6% of monoterpene OH reactivity (Figure 2b), surpassing α-pinene (19.5%) due to its much higher reaction rate with OH (177 × 10^–12^ cm^3^ molecule^–1^ s^–1^) than α-pinene (52.3 × 10^–12^ cm^3^ molecule^–1^ s^–1^).^3^ However, this dominant role of γ-terpinene has received little attention in previous studies. The estimated OH reactivity for isoprene, benzene, styrene, and phenol decreased significantly after correction, with reductions ranging from 13.6% to 69.2% (Figure 4c). The effect was strongest for isoprene, whose OH reactivity decreased from 0.58 to 0.17 s^–1^, corresponding to a 241% overestimation. This overestimation was reduced to 165% during the high-isoprene season, owing to enhanced biogenic emissions. Figure 4d,e compares the total OH reactivity of VOCs measured by PTR-ToF-MS and GC-MS/FID/ECD, derived from the direct-PTR and GC-PTR data sets, respectively. The total VOC OH reactivity was 2.2 s^–1^ for the direct-PTR data set and 1.8 s^–1^ for the GC-PTR data set. Specifically, the contribution of isoprene drops from 26.3% (direct-PTR) to 9.3% (GC-PTR), while those of carbonyls and C_8_–C_9_ aromatics rise from 4.1% to 7.3% and from 6.1% to 9.9%, respectively.

**Figure 4:**
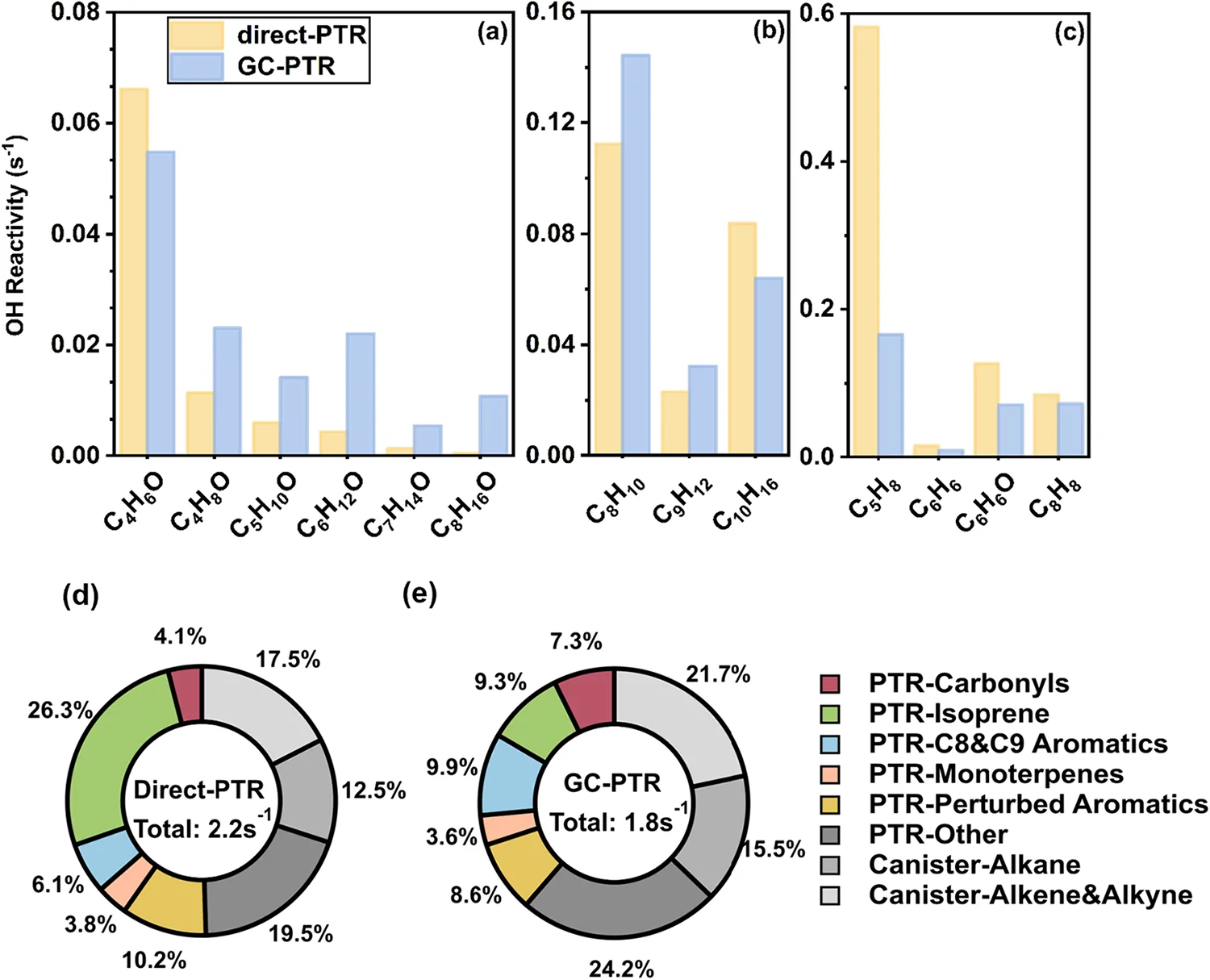
(a–c) Comparison of the OH reactivity estimated by the direct-PTR data set and GC-PTR data set. (d, e) Total OH reactivity of VOCs measured by PTR-ToF-MS and GC-MS/FID/ECD, calculated from the direct-PTR (d) and GC-PTR (e) data sets. The “PTR-Other” group represents other PTR-identified species used in the box model simulation (marked with * in Table S5).

### 3.5 Impacts on Photochemistry Using Box Model Simulation

To further assess how biases in PTR-based VOC quantification affect photochemical interpretations, we conducted box model simulations constrained by different data sets: direct-PTR measurements (BS1) and isomer-resolved GC-PTR measurements (BS2). This comparison allowed us to quantify impacts of unresolved isomer distributions and interference effects on the simulated radical chemistry and ozone production.

As shown in Figure 5a, simulations constrained by the direct-PTR data set (BS1) systematically overestimated daytime production rates of O_3_, RO_2_, and HO_2_ radicals compared with those constrained by the GC-PTR data set (BS2). The daytime-average net O_3_ production rate (Pnet O_3_) decreased from 2.85 ppb/h in BS1 to 1.95 ppb/h in BS2. Similarly, RO_2_ (Pnet RO_2_) and HO_2_ (Pnet HO_2_) production rates declined from 1.46 and 2.27 ppb/h to 0.96 and 1.61 ppb/h, respectively (Figure 5a). The simulated OH and HO_2_ in BS1 and BS2 also followed a similar pattern (Figure S15), with peak OH concentration declining from 4.7 × 10^6^ molecules cm^–3^ in BS1 to 4.5 × 10^6^ molecules cm^–3^ in BS2, and peak HO_2_ concentration declining from 8.6 × 10^7^ molecules cm^–3^ in BS1 to 4.9 × 10^7^ molecules cm^–3^ in BS2. These differences reflect the combined effects of overestimated concentrations for some VOCs, especially isoprene and monoterpenes, and underestimated concentrations for OVOCs in direct-PTR measurements.

**Figure 5:**
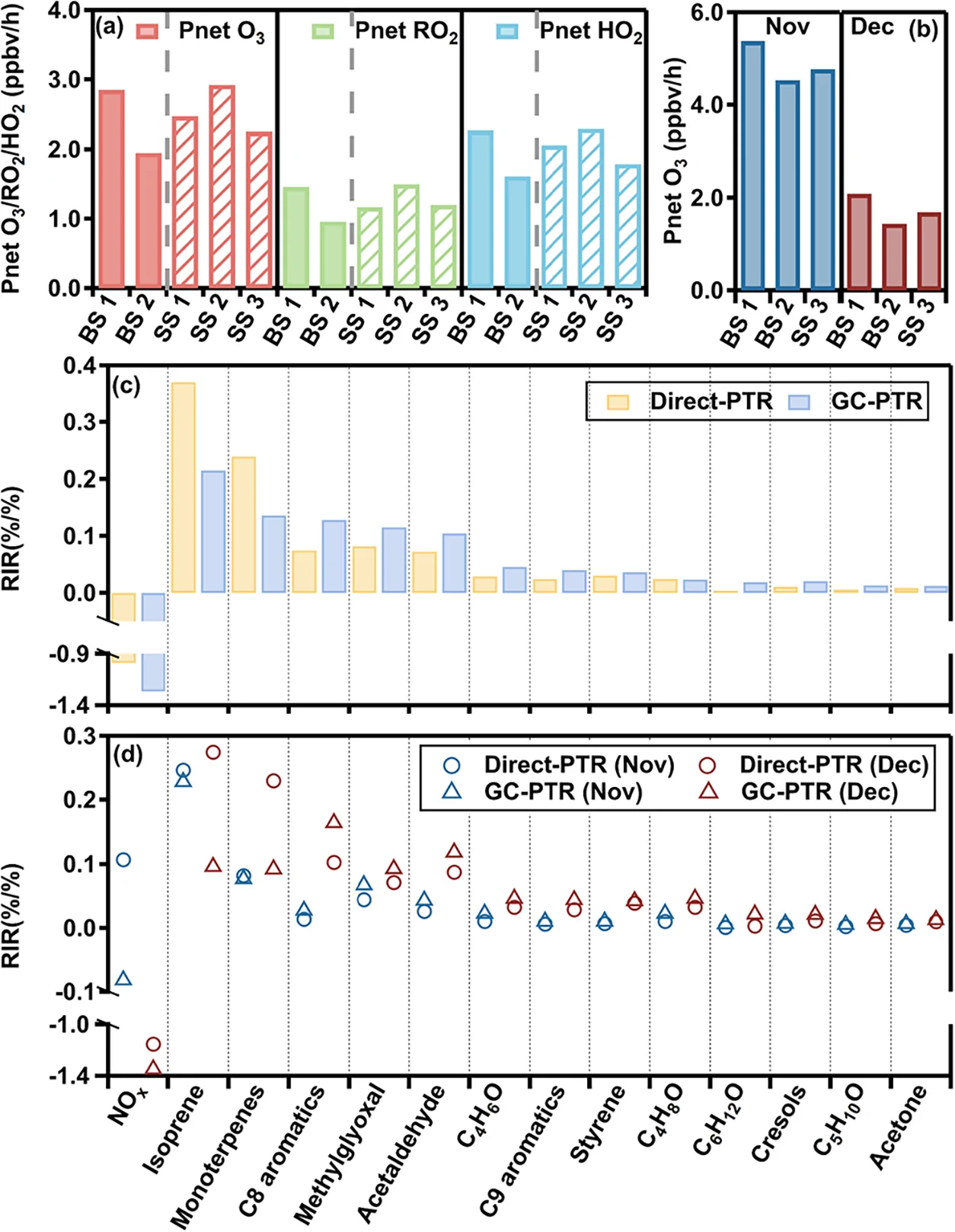
(a) Simulated daytime net production rates of O_3_, RO_2_, and HO_2_ radicals for different scenarios based on direct-PTR and GC-PTR data sets. (b) Simulated daytime net production rates of O_3_ for different scenarios under different seasonal conditions. (c) RIR values of PTR-measured compounds derived from box model simulations using the direct-PTR and GC-PTR data sets over the whole study period. (d) RIR analysis same as (c), but for November and December comparison.

To identify which corrections most strongly influenced model outcomes, three additional sensitivity scenarios were examined (described in the Section 2.5). Incorporating GC-PTR based isomer abundances (SS1) reduced the daytime Pnet O_3_/RO_2_/HO_2_ to 2.47/0.96/2.06 ppb/h. This reduction was primarily driven by the correction of overestimated monoterpenes in the direct-PTR data set, as monoterpenes exhibit substantially higher OH reactivity than other OVOCs and C_8_/C_9_ aromatics that were underestimated in direct-PTR. In contrast, correcting the overestimated benzene, styrene, and phenol mixing ratios for direct-PTR (SS2) led to an increase, rather than a decrease, in Pnet O_3_/RO_2_/HO_2_. This unexpected result is likely because the current MCM v3.3.1 mechanism lacks photolysis pathways for nitrophenol. These species are formed through the reaction of NO_2_ with OH oxidation products of phenol.^46^ Consequently, these nitrated products act as sinks for NO_2_ and radicals, thereby suppressing O_3_ and radical production rates.^47,48^ This could be verified by the negative RIR value for phenol when we estimated the O_3_-precursor sensitivity for individual VOCs.

Correcting the isoprene concentrations alone (SS3) substantially reduced the discrepancy between direct-PTR and GC-PTR scenarios, decreasing the Pnet O_3_/RO_2_/HO_2_ to 2.25, 1.20, and 1.79 ppb/h, respectively. Given that isoprene mixing levels vary significantly across seasons, the fraction of isoprene in the direct-PTR C_5_H_9_^+^ signal also varies seasonally; we further examined the bias under different isoprene conditions. The campaign was divided into two subperiods based on isoprene and O_3_ levels (Figure 3b,c): a high-isoprene period (15–21 November, with a peak diurnal isoprene of 0.57 ppb and O_3_ of 69.0 ppb), and a low-isoprene period (1–19 December, with diurnal peak isoprene of 0.1 ppb and O_3_ of 37.5 ppb). During the high-isoprene period, simulated daytime Pnet O_3_ (Figure 5b) was 5.37 ppb/h (BS1) and 4.53 ppb/h (BS2), indicating that direct-PTR overestimated Pnet O_3_ by 19% (0.84 ppb/h) compared to GC-PTR. This bias is smaller than that observed over the low-isoprene period (0.65 ppb/h, 45%) and the full campaign (46%), but remains significant. Correcting isoprene alone still reduced Pnet O_3_ substantially (from 5.37 to 4.77 ppb/h) in the high-isoprene period. Although the average O_3_ mixing ratio and Pnet O_3_ are lower in winter Hong Kong, previous studies have shown that anthropogenic VOC-driven O_3_ episodes still occur during this season.^5,8^ Quantifying the bias in direct-PTR measurements under such conditions warrants further investigation.

Figure 5c shows the RIR values of the precursors determined from the two base scenario simulations. Consistent with the above OH reactivity estimates, the RIR of isoprene decreased dramatically from 0.37%/% to 0.21%/% after correction. A smaller decrease (0.24 to 0.14%/%) was also found for monoterpene, indicating that the role of biogenic VOCs in O_3_ production is likely overestimated when relying solely on direct-PTR measurements. In contrast, the sensitivities of aromatics (including styrene, C_8_ and C_9_ aromatics) and OVOCs (such as C_4_H_6_O, C_4_H_8_O, C_5_H_10_O, and C_6_H_12_O) were underestimated in direct-PTR-constrained simulations. This underestimation also extended to OVOCs not directly corrected by GC-PTR measurement, including methylglyoxal and acetaldehyde. When constrained by GC-PTR data, isoprene (RIR value: 0.21%/%) and monoterpenes (α-pinene: 0.08%/%, limonene: 0.04%/%, and β-pinene: 0.01%/%) still demonstrated significant sensitivities to O_3_ production, as most influential precursors at this suburban coastal site (Figure S16), which is in alignment with previous studies in suburban Hong Kong.^49–51^ Furthermore, it is important to note that highly reactive monoterpenes such as γ-terpinene are not fully represented in the MCM v3.3.1 mechanism, which may lead to an underestimation of their contributions to O_3_ production and also potentially SOA formation. In addition to C_8_ and C_9_ aromatics, several OVOCs such as methylglyoxal, acetaldehyde, acrolein, cresol, hexanal, and pentanal also exhibited notable RIR values (0.01%/% to 0.12%/%), highlighting their non-negligible roles in regional photochemistry.

The RIR values for NO_*x*_ were −0.99 and −1.27%/% in direct-PTR and GC-PTR simulations, indicating that O_3_ formation at the site was generally VOC-limited. This explains why correcting isoprene and monoterpenes substantially reduced Pnet O_3_. Subperiod analysis (Figure 5d) further revealed that November was characterized by a transition regime, with NO_*x*_ RIR values of 0.10 and −0.08%/% in the direct-PTR and GC-PTR cases, respectively. Under these conditions, isoprene correction had a moderate effect (Pnet O_3_ overestimated by 19%), while December remained VOC-limited (NO_*x*_ RIR: −1.15 and −1.35%/% for direct-PTR and GC-PTR, respectively), resulting in a much larger overestimation (45%). Notably, the less negative NO*_x_* RIR derived from the direct-PTR data set shifted the inferred chemical regime closer to the VOC-NO_*x*_ transition region compared with the GC-PTR data set. Such a shift could bias assessments of O_3_ control strategies by underestimating the effectiveness of VOC emission reductions and overestimating the importance of NO_*x*_ controls.

## 4 Implications for Atmospheric VOCs Analysis

VOCs are major contributors to total OH reactivity and play a pivotal role in driving photochemical pollution processes. PTR-ToF-MS enables rapid, sensitive measurements of a wide range of VOCs and has been extensively employed to investigate their concentrations and atmospheric impacts. However, our results identified two key limitations in conventional PTR-ToF-MS applications that introduce substantial uncertainties into VOC quantification and subsequent photochemical analyses. First, systematic biases arised when quantifying VOC groups composed of multiple isomers, particularly high-carbon-number carbonyls (C number ≥ 5). These biases stemed from isomer-specific ionization and fragmentation patterns thatwere not adequately accounted for in standard PTR-ToF-MS calibration approaches. Second, concentrations of several key VOC species, including isoprene, benzene, styrene, and phenol, were substantially overestimated due to contributions from interference ions produced by other compounds. In this study, interference accounted for approximately 69.2% of the direct-PTR signal assigned to isoprene, 41.8% for benzene, 13.6% for styrene, and 47.1% for phenol.

Several recent studies have utilized PTR-ToF-MS to quantify uncalibrated VOC concentrations and to estimate their contributions to missing OH reactivity and O_3_ production.^19–21,52^ However, uncertainties inherent in PTR-ToF-MS measurements propagate directly into the OH reactivity estimates and photochemical box model simulations. Overestimation of biogenic VOCs, such as isoprene and monoterpenes, can lead to incorrect attribution of OH reactivities and O_3_ formation to natural sources, and can shift the control regime assessment toward the transition region, particularly under low-isoprene, VOC-limited conditions. In contrast, the overestimation is more moderate under high-isoprene, VOC-saturated conditions. At the same time, high-carbon-number carbonyls, which are often uncalibrated, play a much larger role in OH reactivity and O_3_ production than previously recognized.^8^ In this study, the OH reactivities of these carbonyls were underestimated by 57% to 95%, a discrepancy that may help bridge the gap between measured and modeled total OH reactivity reported in a previous study.^20^ Model simulations further indicate that the sensitivity of O_3_ production to C_5_ and C_6_ carbonyls was underestimated by 55% and 89%, respectively.

Beyond biases in absolute concentrations, the lack of isomer-resolved measurements limits accurate assessment of VOC precursor sensitivities and hampers the development of effective emission control strategies. Differences in chemical reactivity among isomers can strongly influence OH loss rates and O_3_ formation pathways, yet these differences are obscured when VOCs are treated as single, unresolved species. Taken together, these findings highlight the need for improved characterization of VOC isomer distributions, compound-specific calibration factors, and robust correction of interference effects in PTR-ToF-MS measurements. Such improvements are essential for accurately quantifying the contributions of VOC precursors to photochemical pollution and for designing effective strategies to control O_3_ formation.

## Supplementary Material



## Notes

Isomer identification for GC-PTR; calibration factor determination for isomers; photochemical box model settings; interference correction method; description for OH reactivity estimation for VOCs; diagram of GC-PTR system; GC-PTR chromatogram; diurnal profiles of model constraints; interference contributions; diurnal variation of isomers fraction; isoprene correction comparisons; diurnal profiles of simulated OH and HO_2_ radical; RIR value for individual species; list of identified compounds in GC-PTR sample; calibration factors for identified compounds; and model-constrained VOCs (PDF)
