# Supplementary material for: Photochemical Impacts from Resolving Isomers and Interference Biases in PTR-ToF-MS Measurements of Atmospheric VOCs
Source: Environ Sci Technol. 2026 Aug 5;60(32):22836–48. doi: 10.1021/acs.est.6c08699 (PMC13492273; doi:10.1021/acs.est.6c08699)
Supplement: Supplementary file 1 [file es6c08699_si_001.pdf]

Supporting information for

Photochemical Impacts from Resolving Isomers and Interference Biases in PTR-ToF-MS Measurements of Atmospheric VOCs

Xin Feng[1], Lirong Hui[1], Yi Chen[1,2], Penggang Zheng[1,3], Megan S. Claflin[4], Brian M. Lerner[4], Yao Chen[1], Jiali Zhong[1], Dasa Gu[1], Yang Xu[1], Yijie Yao[1], Mengyuan Chu[1], Jia Guo[5], Qi Ying[1] Jian Zhen Yu[1,2], Douglas Worsnop[4], and Zhe Wang[1,*]

[1] *Division of Environment and Sustainability, The Hong Kong University of Science and Technology, Hong Kong SAR, 999077, China*

[2] *Department of Chemistry, The Hong Kong University of Science and Technology, Hong Kong SAR, 999077, China*

[3] *School of the Environment and Sustainable Engineering, Eastern Institute of Technology, Ningbo, 315200, China.*

[4] *Aerodyne Research, Inc., Billerica, Massachusetts 01821, USA*

[5] *Environmental Central Facility, The Hong Kong University of Science and Technology, Hong Kong SAR, 999077, China*

[*]Correspondence to: Zhe Wang: z.wang@ust.hk

Text S1. Isomer identifications

Table S1 presents the retention times (RTs) determined by GC-Vocus calibration and the corresponding retention indices (RIs) from the NIST database (https://webbook.nist.gov/). Non-standard species were identified by matching their observed RTs with RIs derived from the linear fit of RTs and RIs in Figure S2. For example, three individual peaks were found in the chromatogram of $C_8H_{10}H^+$, with the retention times of 361.4s, 367.0s, and 387.7s (Figure S3). The species eluted at 367.0s was assigned to be m-xylene based on GC-PTR calibration. The other two peaks, eluting before and after m-xylene (RI: 855.5), likely correspond to ethylbenzene and o-xylene, with estimated RTs of 360s (RI: 850) and 386s (RI: 890), respectively. Therefore, these two peaks were assigned to ethylbenzene and o-xylene. Additionally, another corresponding peak of $C_6H_7^+$ was found for ethylbenzene, consistent with previous studies that the ethylbenzene will fragments to $C_6H_7^+$ in PTR[1], which could serve as additional supporting evidence for the identification of ethylbenzene. The p-xylene is collected with m-xylene for MXT-624 column. Following this approach, we identified 7 alkenes, 11 aromatics, 14 aldehydes, and 14 ketones. Details can be found in Table S2.

The identification of pentanal in GC-PTR chromatogram required special handling. Although pentanal theoretically generates a $C_5H_{11}O^+$ signal, no discernible pentanal peak was observed in the corresponding chromatogram. Instead, its characteristic fragmentation signature appeared in the $C_5H_9^+$ channel (Figure S4), attributed to two key factors: (1) near-coelution of pentanal (RI=668) and 3-pentanone (RI=669) under the analytical conditions, and (2) extensive fragmentation of pentanal during ionization process. Thus, the $C_5H_{11}O^+$ from pentanal is masked by the strong signal from 3-pentanone. Post-campaign calibration of GC-PTR using standard gas with pentanal revealed a 21:1 peak area ratio between the $C_5H_9^+$ fragments and $C_5H_{11}O^+$ signal. Consequently, quantitative analysis required deriving pentanal signal in $C_5H_{11}O^+$ through fragmentation-correction calculations of $C_5H_9^+$ signal rather than direct peak integration.

Text S2. Calibration factors determination

The correction factor (***f***) is defined as the ratio of the chosen quantitative ion in the total ionization signals (the sum of quantitative ion and additional ions) and is calculated by the following formula:

$$f = \frac{Signal_{Quan}}{Signal_{Quan} + Signal_{Add}} \tag{1}$$

where $Signal_{Quan}$ represents the signal intensity of the quantitative ions, and $Signal_{Add}$ denotes the combined signal from all additional product ions. This factor was quantified using peak areas of the identified compounds in GC-PTR chromatograms of quantitative and additional product ions signals. Subsequently, for the calibrated species, the revised sensitivity ($Sen_{Revised}$) was calculated by formula (2), and employed to establish the k_{PTR} and sensitivities relationship.

$$Sen_{Revised} = \frac{Cal_{Calibration}}{f} \tag{2}$$

Where $Cal_{Calibration}$ represents the calibration factors determined by the gas standard in RT-PTR calibration. for the quantitative ion. As demonstrated in Figure S5, this correction significantly enhanced sensitivity values corresponding to the specific k_{PTR}, with the regression slope increasing from 2899 to 3278.

More importantly, the *f* value could also be applied to determine the calibration factors for the non-standard species. The calibration factor (Cal_{Corr}) was determined by formula (3):

$$Cal_{Corr} = Sen_{k_{PTR}} \times f \quad (3)$$

where $Sen_{k_{PTR}}$ represents the sensitivity derived from the k_{PTR} and revised k_{PTR}-sensitivity regression. Details about *f* and Cal_{Corr} for non-calibrated compounds are provided in Table S4. The transmission efficiency was also determined based on the fitting function in Figure S6.

Text S3. Photochemical Box Model

The model pre-ran for 5 days to ensure the stabilized unconstrained species concentration in the system, and output on the sixth day was taken for further analysis. The simulated organic peroxyl (RO_2), hydroperoxyl (HO_2), and OH radicals were employed to calculate the net O_3 production rate according to formula 4.

$$\begin{aligned} P(O_3) = {} & k_{HO_2+NO}[HO_2][NO] + \sum_i (k^i_{RO_2+NO}[RO^i_2])[NO] \\ & - k_{O(^1D)}k_{H_2O}[O(^1D)][O_3] - k_{HO_2+O_3}[HO_2][O_3] - k_{OH+O_3}[OH][O_3] \\ & - k_{OH+NO_2}[OH][NO_2] - \sum_j \left(k^j_{alkene+O_3}[alkene^j]\right)[O_3] \\ & - \sum_i (k^i_{RO_2+NO_2}[RO^i_2])[NO_2] - 2\sum_l (k^l_{VOCs+NO_3}[VOCs^l])[NO_3] \end{aligned} \quad (4)$$

Where *k* constants are the reaction rate coefficients for their subscript reactions, *i, j, l* refer to the number of species of RO_2, alkene, and VOCs react with NO, NO_2 and NO_3, respectively[2].The relative incremental reactivity (RIR) was also calculated to investigate the sensitivity of O_3 production rate to the VOCs precursors in GC-PTR and direct-PTR data set. RIR is defined as the ratios of the decrease in net O_3 production in daytime (07:00 -19:00 LST) to the reduction in O_3 precursors.[3-5] The calculation of RIR follows formula 5.

$$RIR(x) = \frac{\Delta P(O_3)/P(O_3)}{\Delta C(x)/C(x)} \quad (5)$$

Where *x* represents the specific O_3 precursors (i.e., NO_x, VOCs), the $\Delta P(O_3)/P(O_3)$ is the percentage change in net O_3 production attributed to the reduction of the precursors (*x*), and the $\Delta C(x)/C(x)$ is the percentage change of *x* concentration (10% reduction in this study). Detailed information could be found in previous study.[6-8]

Text S4. Correction of interference

This correction approach uses the isomer-resolved direct-PTR signals to quantify the abundance of interfering species and applies interference-to-quantitative-ion ratios obtained from GC-PTR chromatograms to estimate their contributions to the target PTR ion signals. The corrected concentration of a given direct-PTR ion is expressed as:

$$C_xH_yO_z{}^+{}_{corrected} = C_xH_yO_z{}^+{}_{PTR} - \sum \frac{C_xH_yO_z{}^+{}_{interference,\ GC}}{MH^+{}_{interference,\ GC}} \times MH^+{}_{interference,RT} \quad (6)$$

where $C_xH_yO_z{}^+{}_{PTR}$ is the direct-PTR measured signal of target ion, $\frac{C_xH_yO_z{}^+{}_{interference,\ GC}}{MH^+{}_{interference,\ GC}}$ represent the ratio of interference ions and corresponding quantitative ion of interference compounds determined from GC-PTR measurement, and $MH^+{}_{\text{interference, RT}}$ represents the direct PTR signal of the interfering compounds.

The interference signal of $C_5H_9^+$ was contributed by three long-chain aldehydes (pentanal, octanal, and nonanal) and unknown $C_9H_{19}^+$. The signal contributed by pentanal and octanal has been resolved from the direct-PTR signals of $C_5H_{11}O^+$ and $C_8H_{17}O^+$ signal in Section 3.1. The $C_9H_{19}O^+$ signal was predominated by nonanal in PTR measurement. While the signal of $C_9H_{17}^+$ was contributed by the fragments of nonanal, and the RT signal of unknown $C_9H_{17}^+$ was determined by subtracting the contribution from nonanal following formula (7).

$$unknown\ C_9H_{17}{}^+{}_{RT} = C_9H_{17}{}^+{}_{PTR} - \frac{C_9H_{17}{}^+{}_{Nonanal,GC}}{C_9H_{19}O^+{}_{Nonanal,GC}} \times C_9H_{19}O^+{}_{PTR} \quad (7)$$

The fragmentation ratio of nonanal ($C_9H_{17}^+$/ $C_9H_{19}O^+$) could be determined by GC-PTR chromatogram. Then the isoprene could be determined by subtracting the contribution from the interference ions at $C_5H_9^+$ using formula (8).

$$C_5H_9{}^+{}_{isoprene} = C_5H_9{}^+{}_{PTR} - \sum \frac{C_5H_9{}^+{}_{Interference,GC}}{MH^+{}_{Interference,GC}} \times MH^+{}_{RT} \quad (8)$$

Here, the interference $C_5H_9^+$ signal was determined by converting the resolved quantification ions signals of interferences (MH^+_{RT}) to their $C_5H_9^+$ signal using the $C_5H_9^+$/ MH^+ ratios in GC chromatogram. Similarly, the signals of $C_6H_7^+$, $C_8H_9^+$, and $C_6H_6O^+$ in direct-PTR could be corrected using the following formulas:

$$C_6H_7{}^+{}_{Benzene} = C_6H_7{}^+{}_{PTR} - C_8H_{11}{}^+{}_{Ethylben,RT} \times \frac{C_6H_7{}^+{}_{Ethylben,GC}}{C_8H_{11}^+{}_{Ethylben,GC}} - C_7H_7O^+{}_{PTR} \times \frac{C_6H_7{}^+{}_{Benzaldhyde,GC}}{C_7H_7O^+{}_{Benzaldhyde,GC}} \quad (9)$$

$$C_8H_9{}^+{}_{Styrene} = C_8H_9{}^+{}_{PTR} - C_8H_{11}{}^+{}_{m\&p-Xylene,RT} \times \frac{C_8H_9{}^+{}_{m\&p-Xylene,GC}}{C_8H^+_{11\,m\&p-Xylene,GC}} \quad (10)$$

$$\begin{aligned} C_6H_7O^+{}_{Phenol} = {} & C_6H_7O^+{}_{PTR} - C_6H_7{}^+{}_{Benzene} \times \frac{C_6H_7O^+{}_{Benzene,GC}}{C_6H_7{}^+{}_{Benzene,GC}} \\ & - C_7H_9{}^+{}_{PTR} \times \frac{C_6H_7O^+{}_{Toluenen,GC}}{C_7H_9{}^+{}_{Toluene,GC}} - C_8H_{10}{}^+{}_{Ethylben,RT} \times \frac{C_6H_6O^+{}_{Ethylben,GC}}{C_8H_{11}{}^+{}_{Ethylben,GC}} \\ & - C_7H_7O^+{}_{PTR} \times \frac{C_6H_7O^+{}_{Benzaldhyde,GC}}{C_7H_7O^+{}_{Benzaldhyde,GC}} \quad (11) \end{aligned}$$

Here the signal of $C_7H_9^+$ ($C_7H_9{}^+{}_{PTR}$) and $C_7H_7O^+$ ($C_7H_7O^+{}_{PTR}$) in direct-PTR is mainly attributed to toluene and benzaldehyde and directly used for correction. The signal of ethylbenzene ($C_8H_{11}{}^+{}_{Ethylben,RT}$) and m&p-xylene ($C_8H_{11}{}^+{}_{m\&p-Xylene,RT}$)were determined by the resolved $C_8H_{11}^+$ RT-PTR signal in Section 3.1.

Text S5. OH reactivity

To assess the influence of different VOC species on the loss of hydroxyl (OH) radicals, the OH reactivities of isomers (L_{OH}) were estimated using the following equation [9, 10]

$$OH\ Reactivity = [VOC]_i \times k^i_{OH} \quad (12)$$

Here, $[VOC]_i$ represents the concentration of individual VOCs (molecule/cm^3), and k^i_{OH} is the reaction rate constant for individual VOCs reacting with OH at 298 K (cm^3/(molecule·s)), obtained from previous studies.[11-13] The k_{OH} values for compounds in the direct-PTR data set were determined by possible dominated isomers or the average of all isomers following previous PTR measurement.[14, 15] For instance, the C_4H_8O was treated solely as MEK.

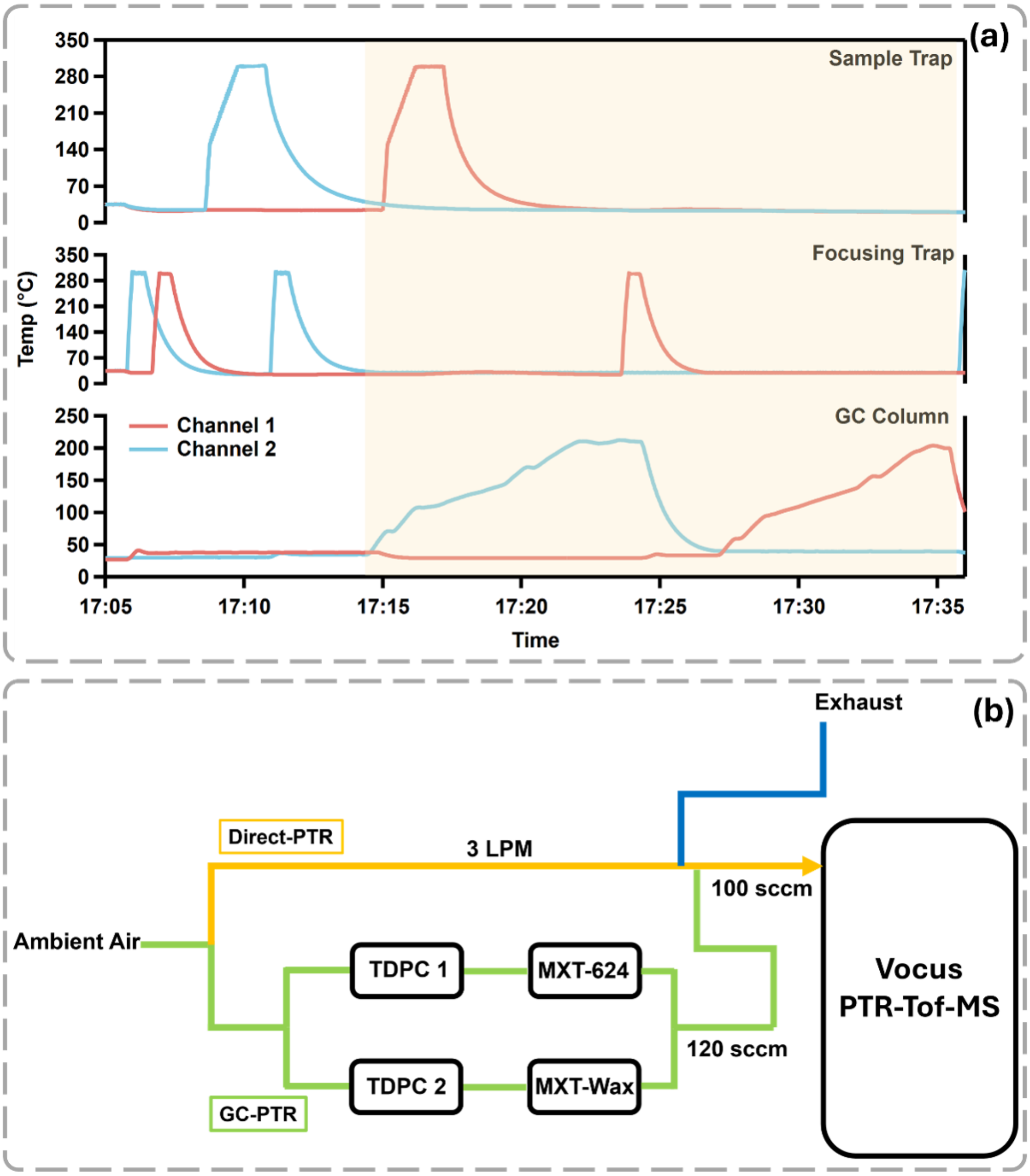

Figure S1. (a) Temperature profile for the two-channel GC system. The shaded area indicates the elution period from the GC column and PTR data acquisition period. (b) Instrument diagram for GC-PTR (green) and direct-PTR (orange) mode.

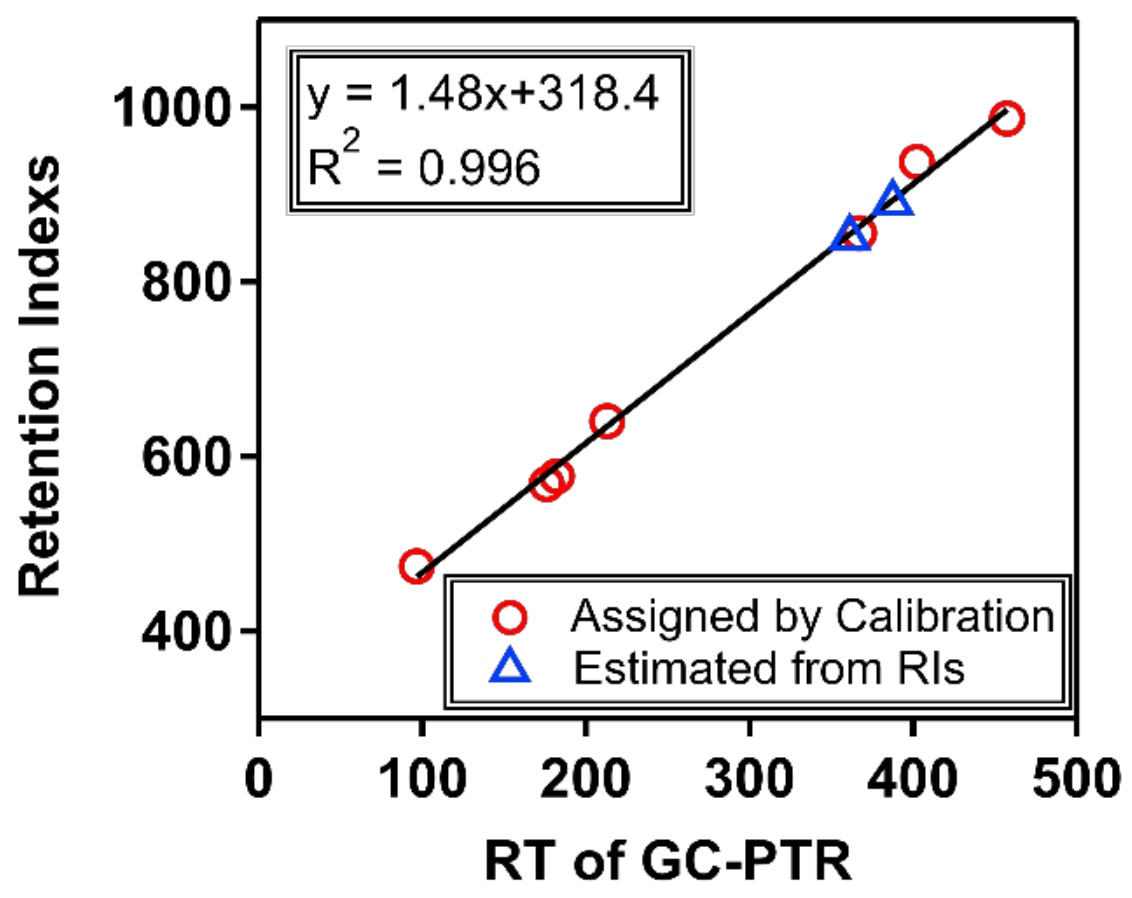

Figure S2. Relationships between the RTs identified by GC-PTR calibration and the corresponding RIs from NIST database. The red circles represent the compounds identified during GC-PTR measurement, and the blue triangles represent ethylbenzene and o-xylene.

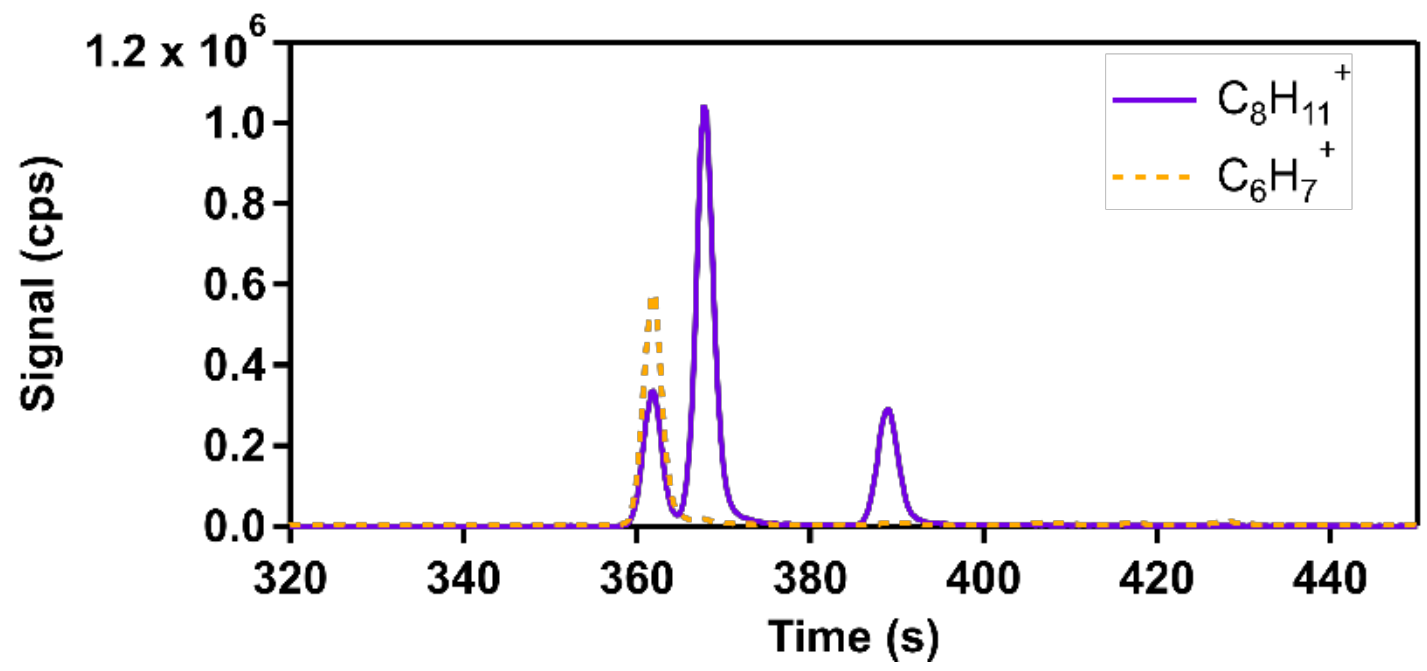

Figure S3. Chromatogram of $C_8H_{11}^+$ for ambient sample in GC-PTR mode.

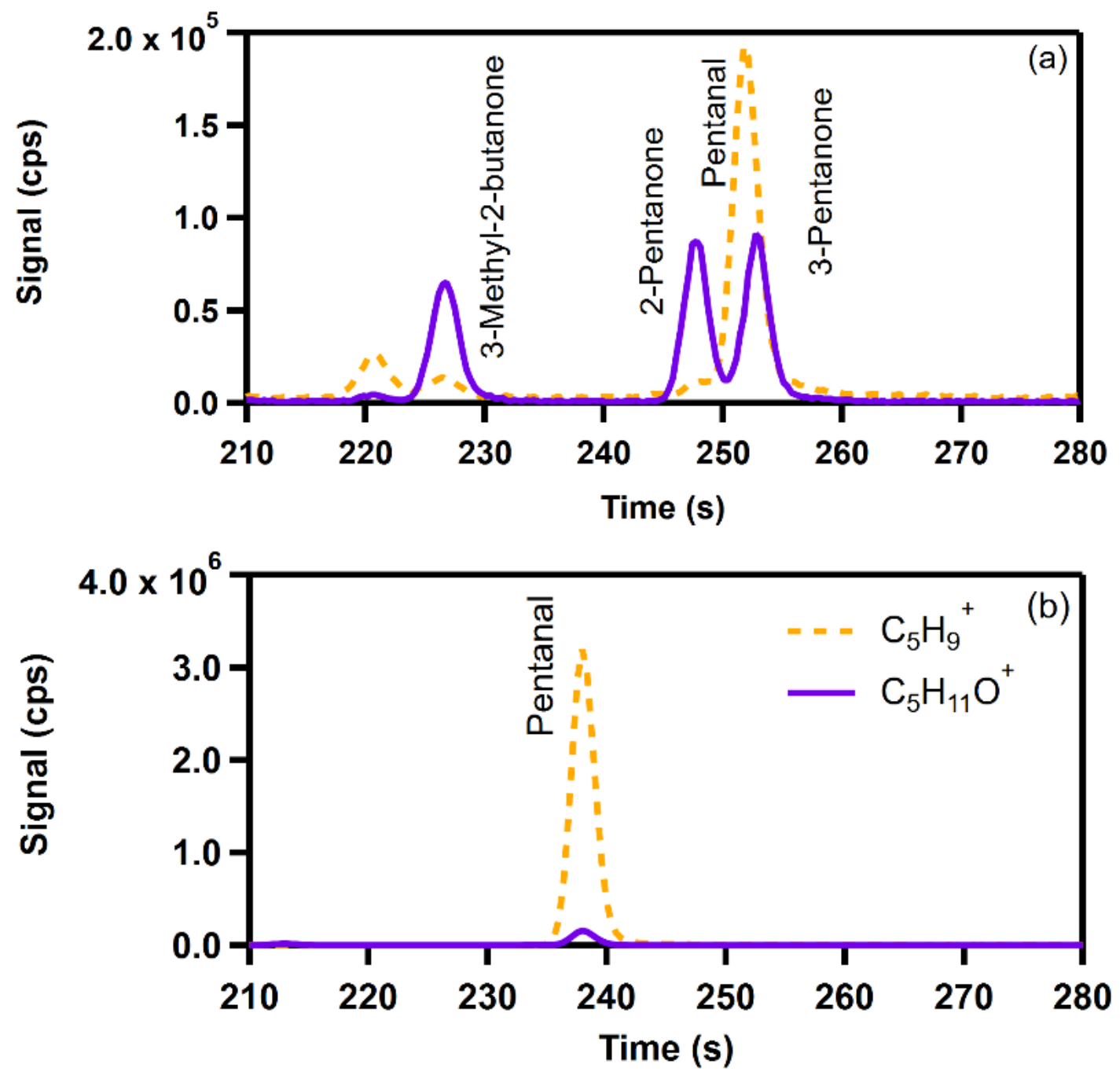

Figure S4. Chromatograms of $C_5H_{11}O^+$ and $C_5H_9^+$ for (a) ambient sample and (b) pentanal calibration at post-campaign calibration. The shift in pentanal RT in panel (b) is due to the recovery of the GC column temperature stability following heater maintenance.

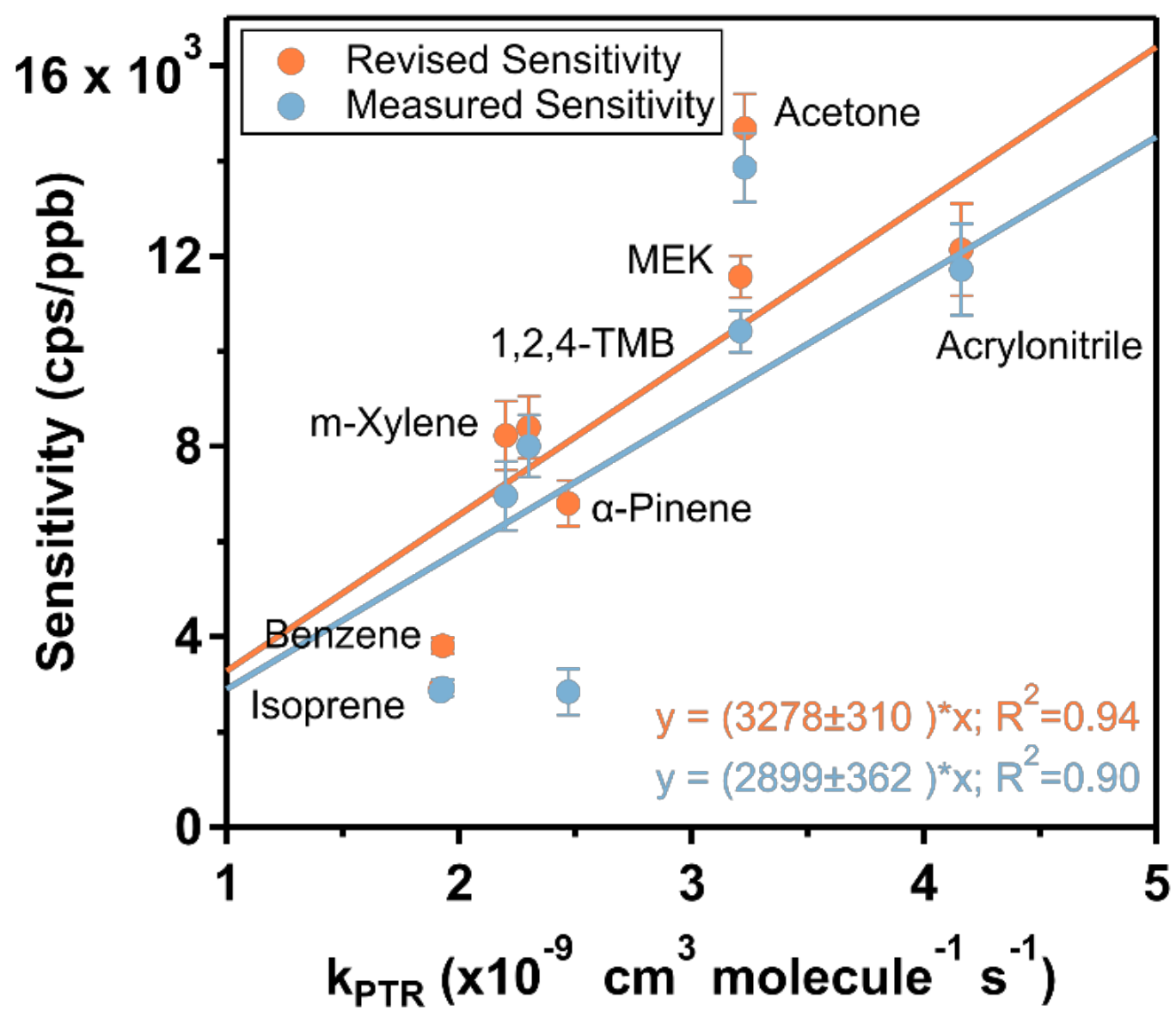

Figure S5. Correlation between PTR sensitivity and proton-transfer reaction rate coefficients (k_{PTR}). Experimental (blue) and revised (orange) sensitivity- k_{PTR} relationships are shown with respective regression lines.

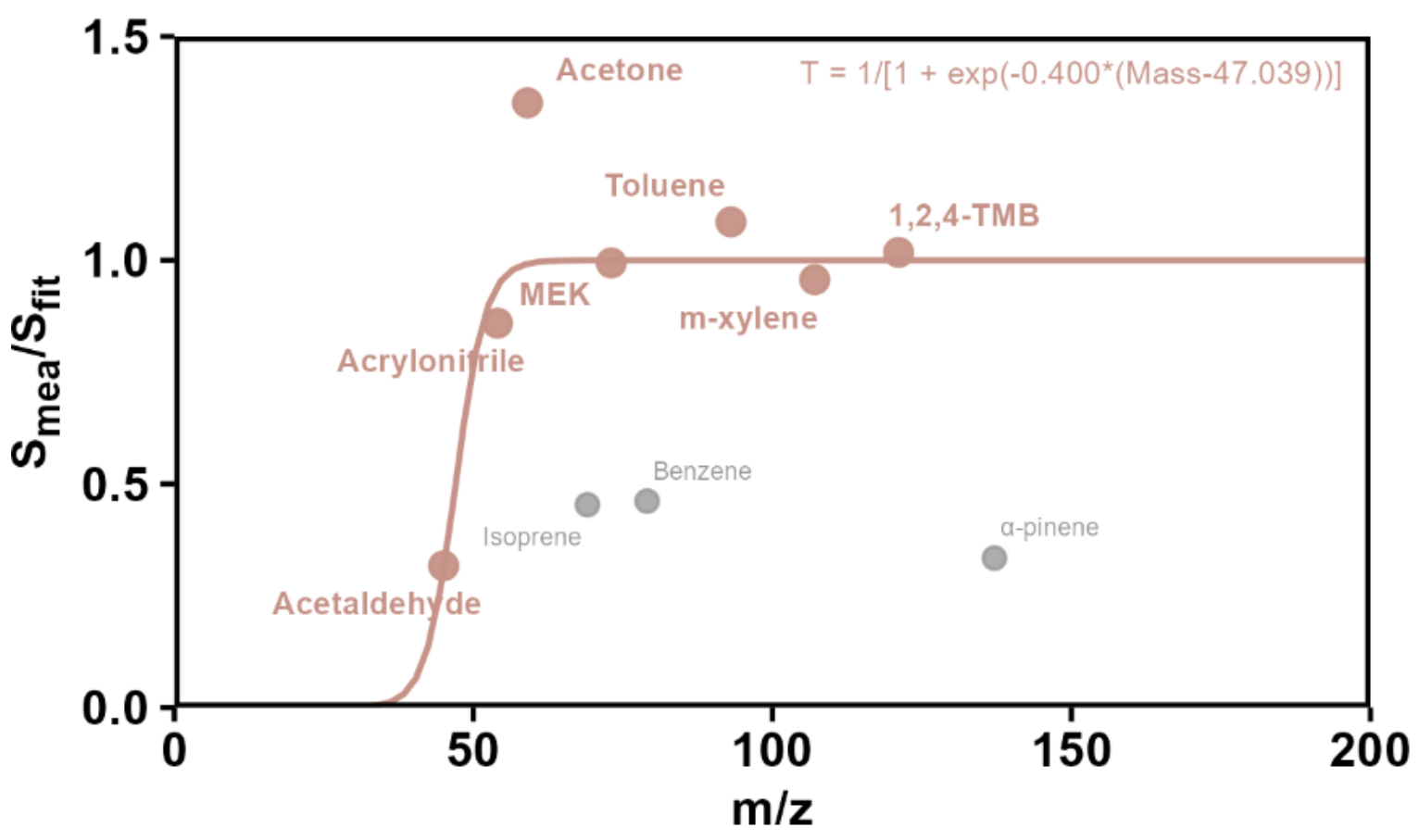

Figure S6. The transmission efficiency of gas standards. The species marked in gray are not included in transmission efficiency estimation due to the additional ions production in Vocus.

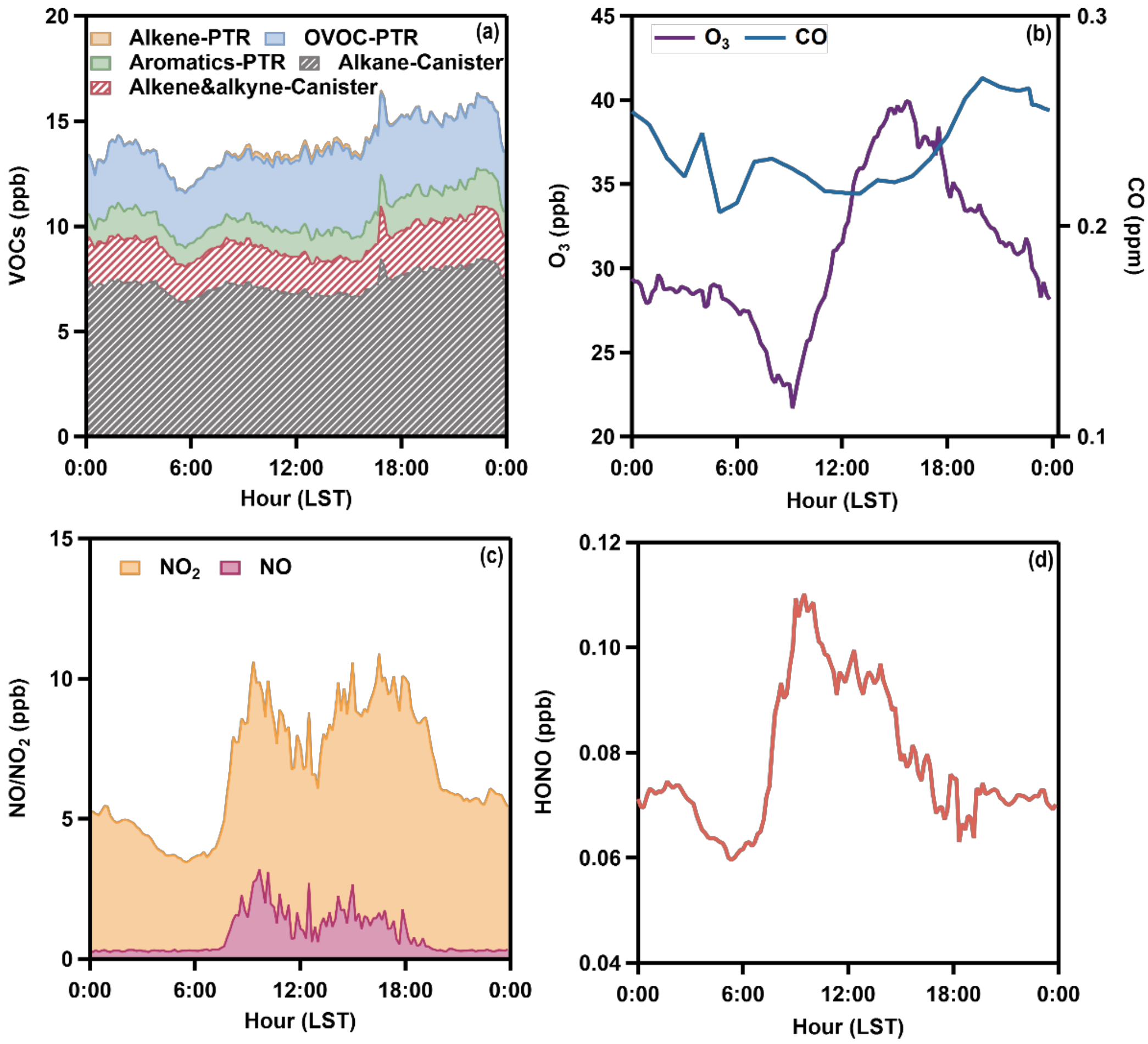

Figure S7. Diurnal profiles of the mixing ratios of sum VOCs, O_3, CO, NO, NO_2, and HONO constrained in BS2.

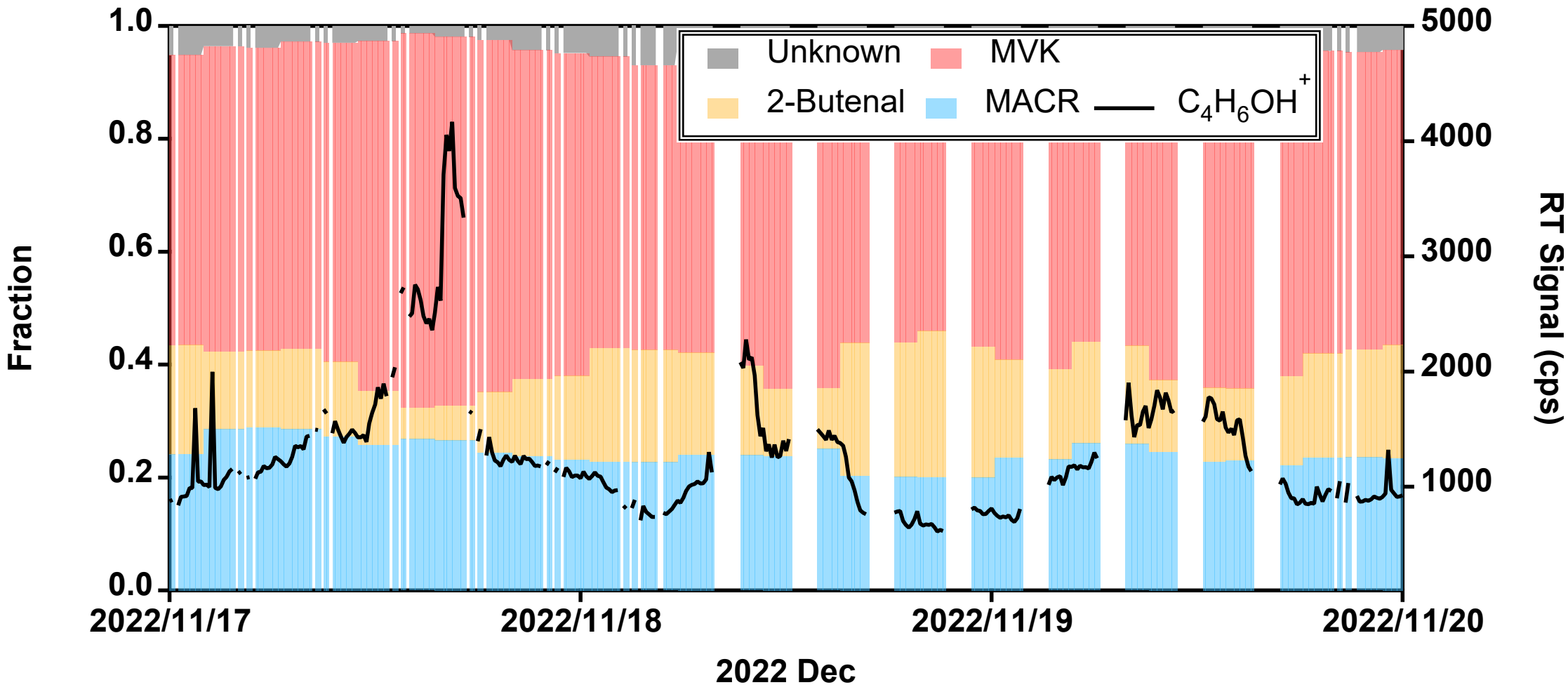

Figure S8. The time series of $C_4H_7O^+$ signal measured by the direct PTR with contributing fractions of MVK, MACR, and 2-butanal, resolved by the GC-PTR, to the total signal.

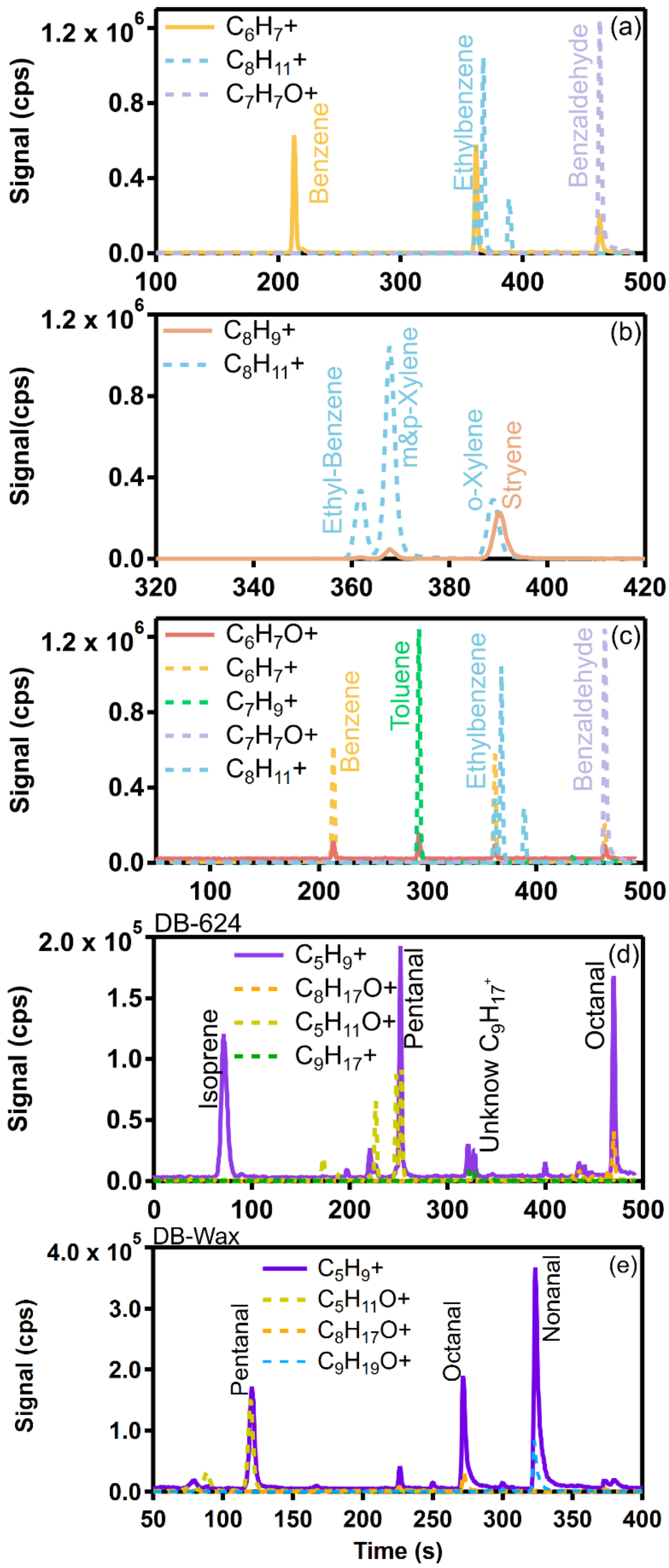

Figure S9. GC chromatograms of the ambient sample, showing the contribution of interference to the signal of (a) $C_6H_7^+$, (b) $C_8H_9^+$, (c) $C_6H_7O^+$, (d-e) $C_5H_9^+$. The nonanal was identified at the DB-Wax Channel.

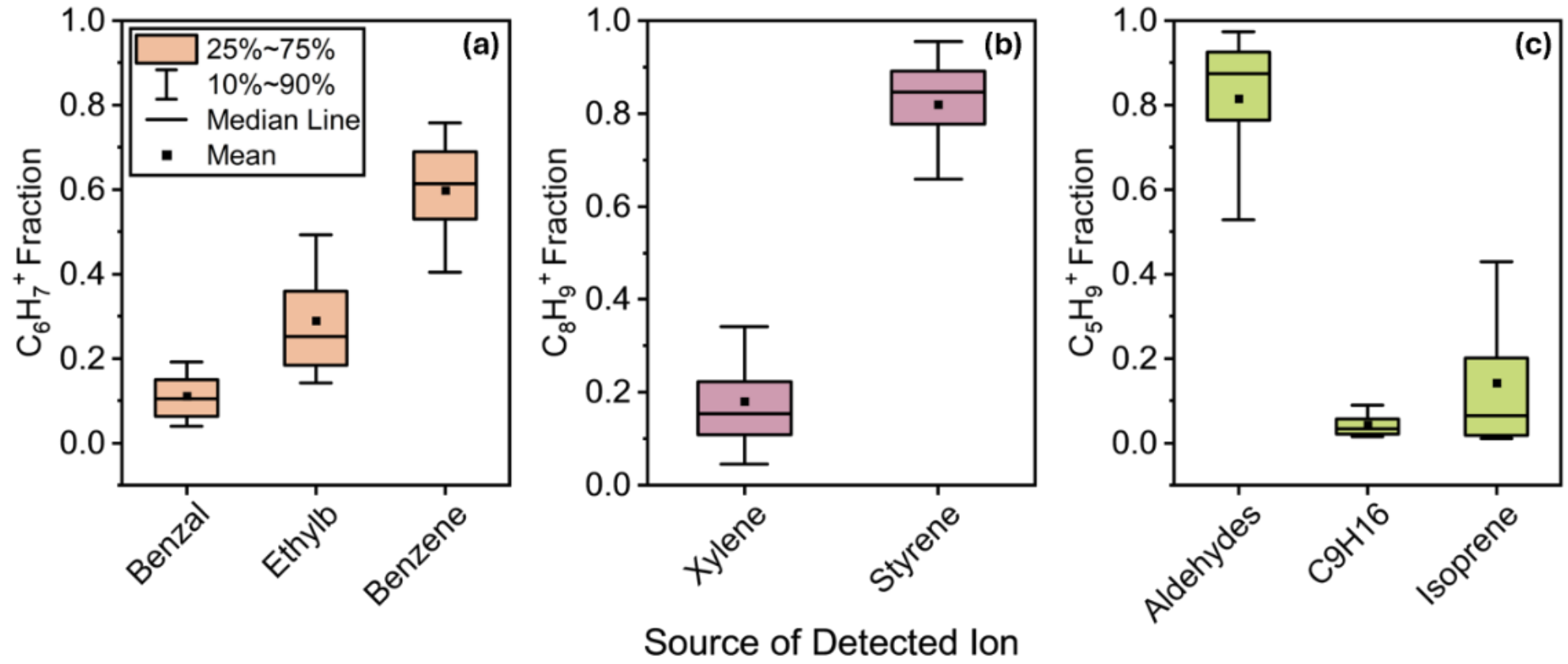

Figure S10. Fraction of the interference peak areas and quantified compounds peak areas determined from GC-PTR chromatogram.

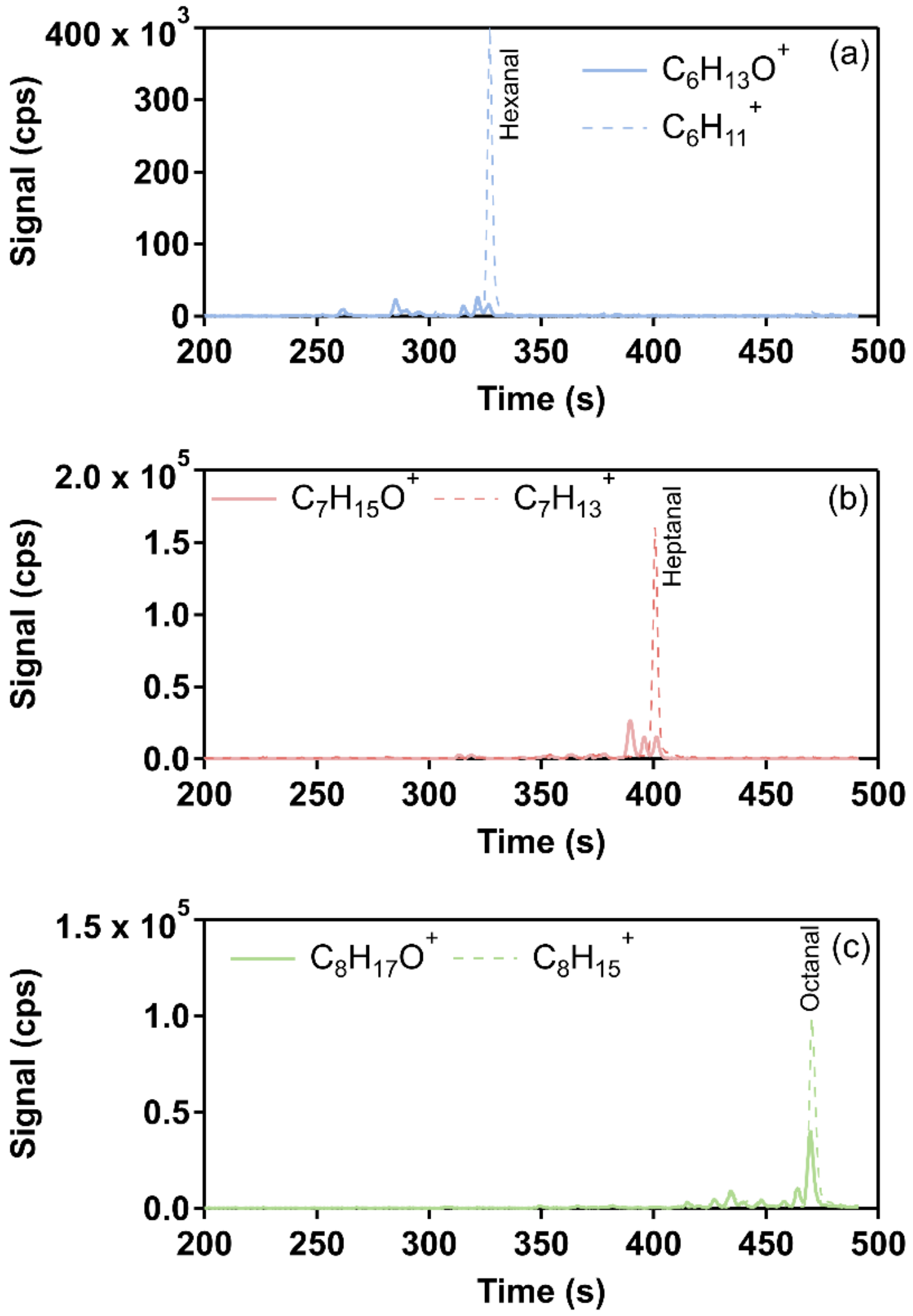

Figure S11. Exemplary ambient chromatogram of $C_6H_{13}O^+$, $C_7H_{15}O^+$, and $C_8H_{17}O^+$.

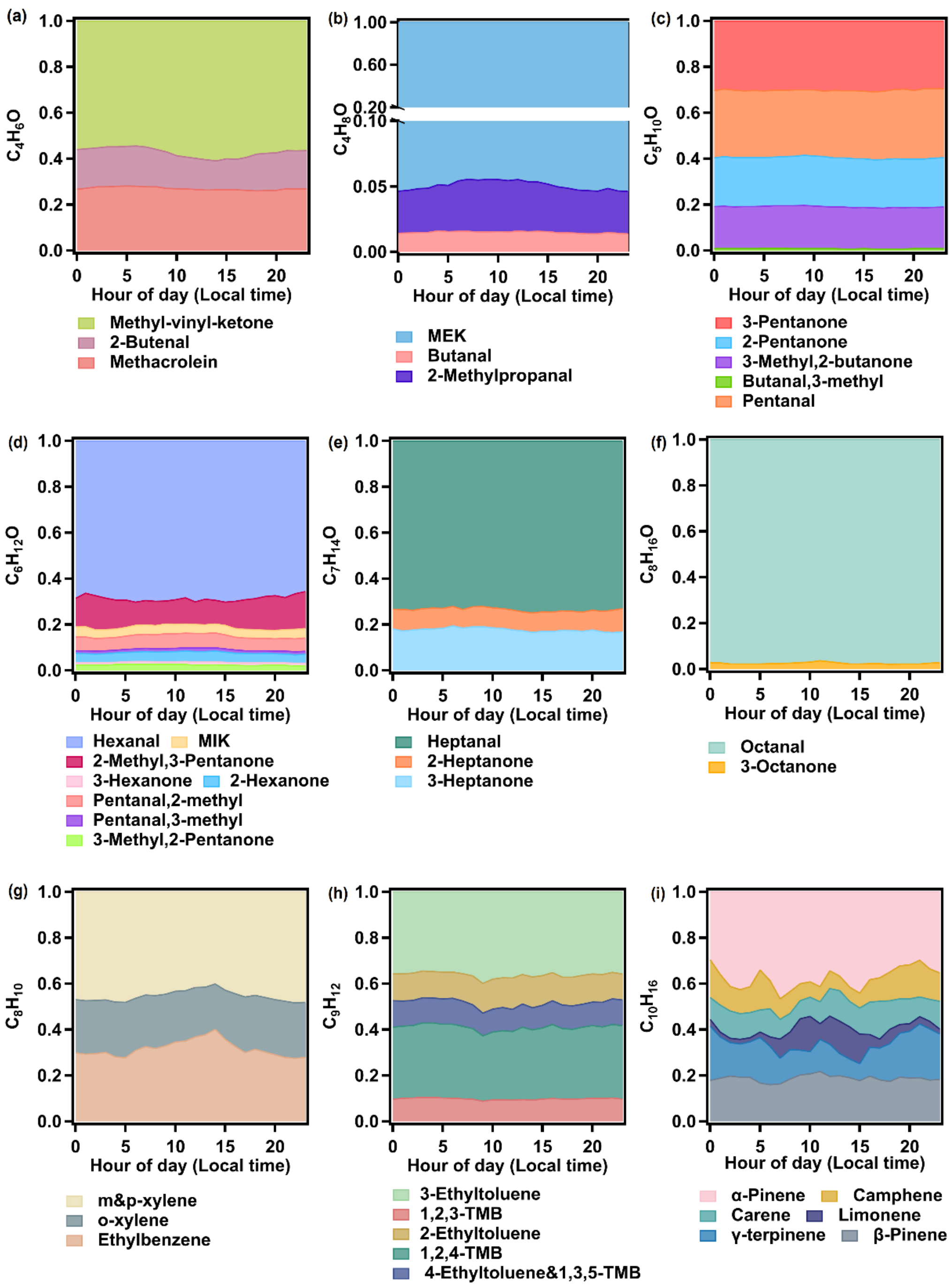

Figure S12.Diurnal variation of isomers concentration fraction determined by GC-PTR data set.

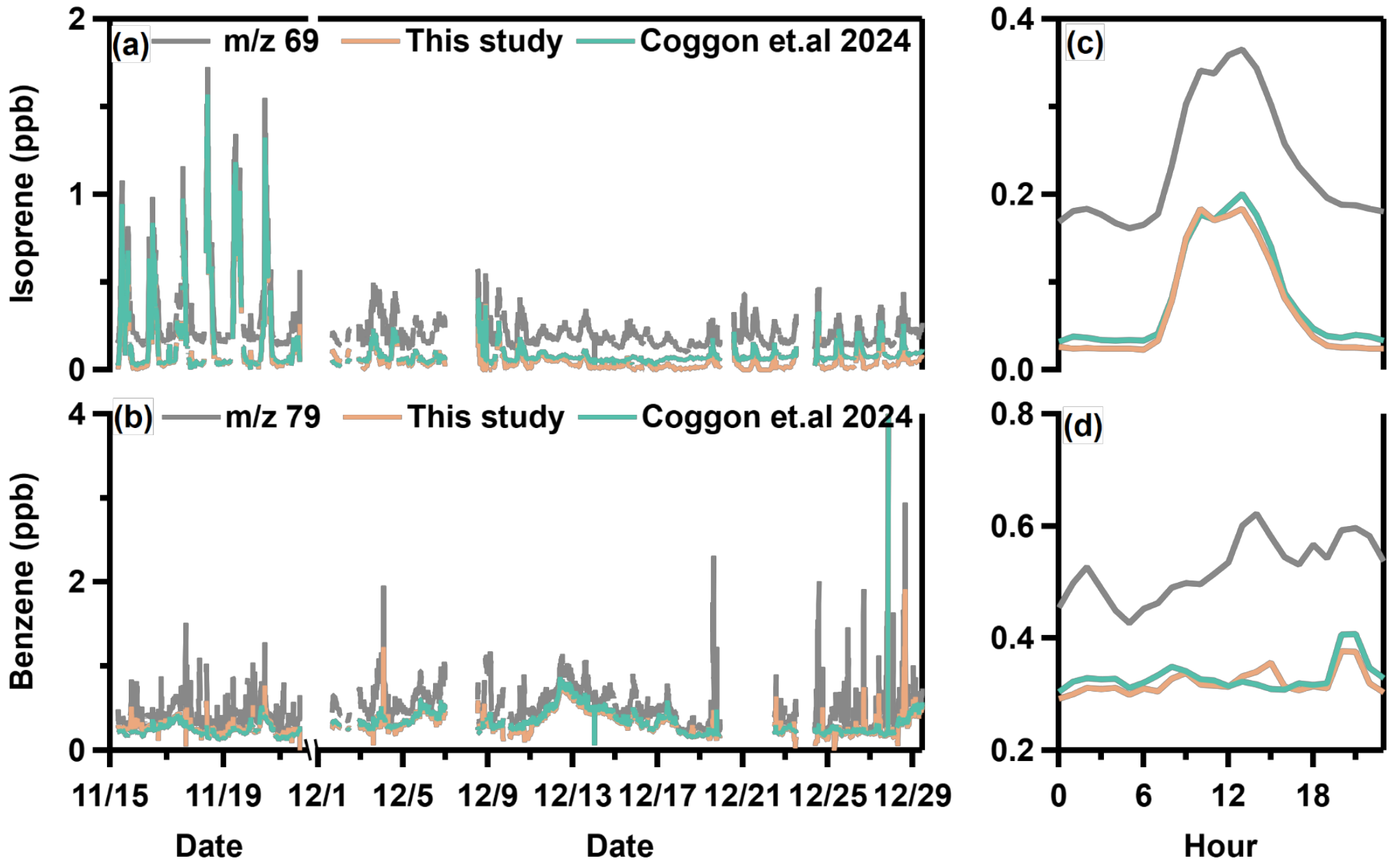

Figure S13. Comparison between the time series and diurnal of isoprene and benzene concentration determined by direct-PTR signals, corrected by our study and Coggon et al.[16].

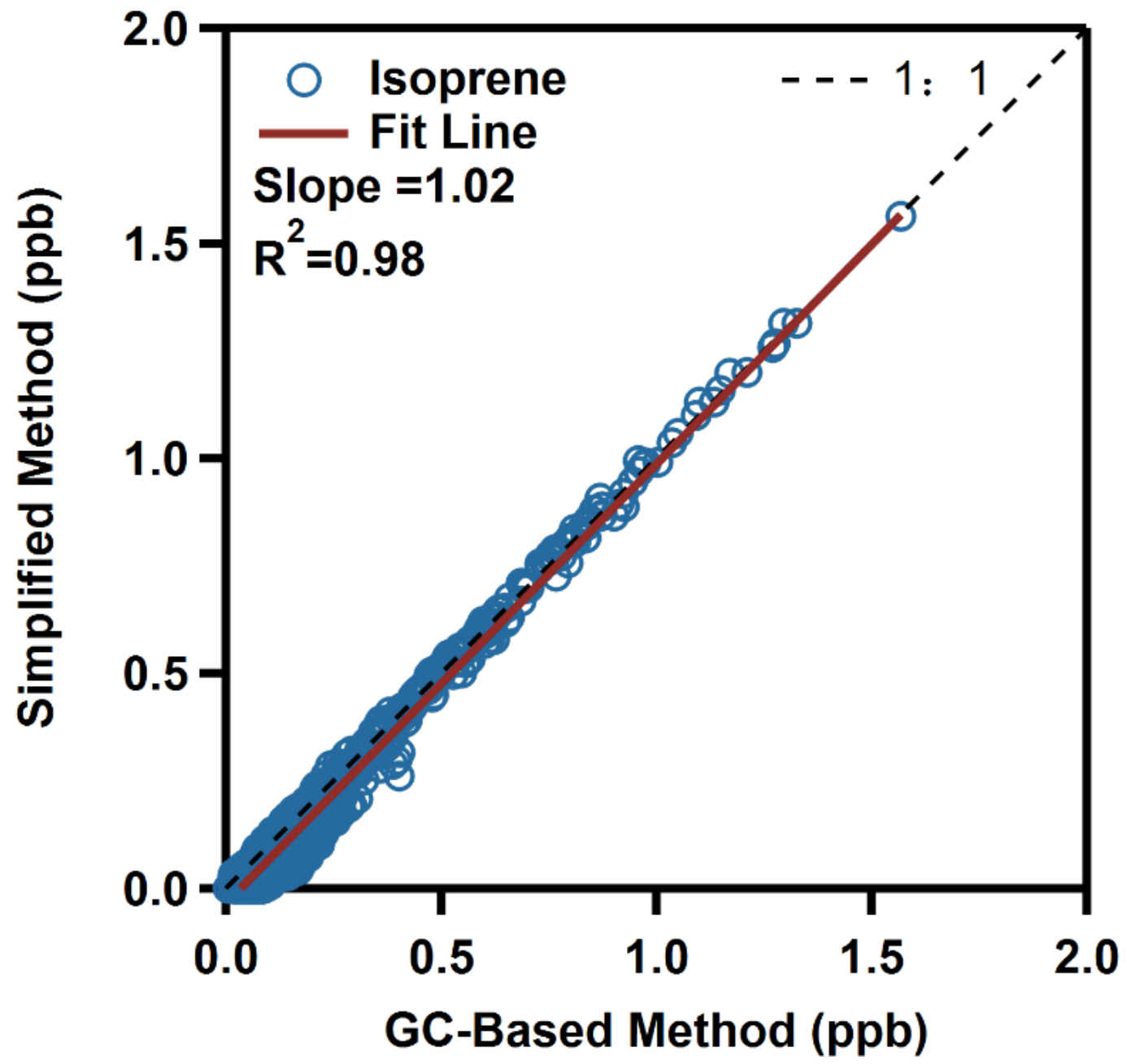

Figure S14.Correlation between the isoprene corrected by GC-based method and the simplified method in this study.

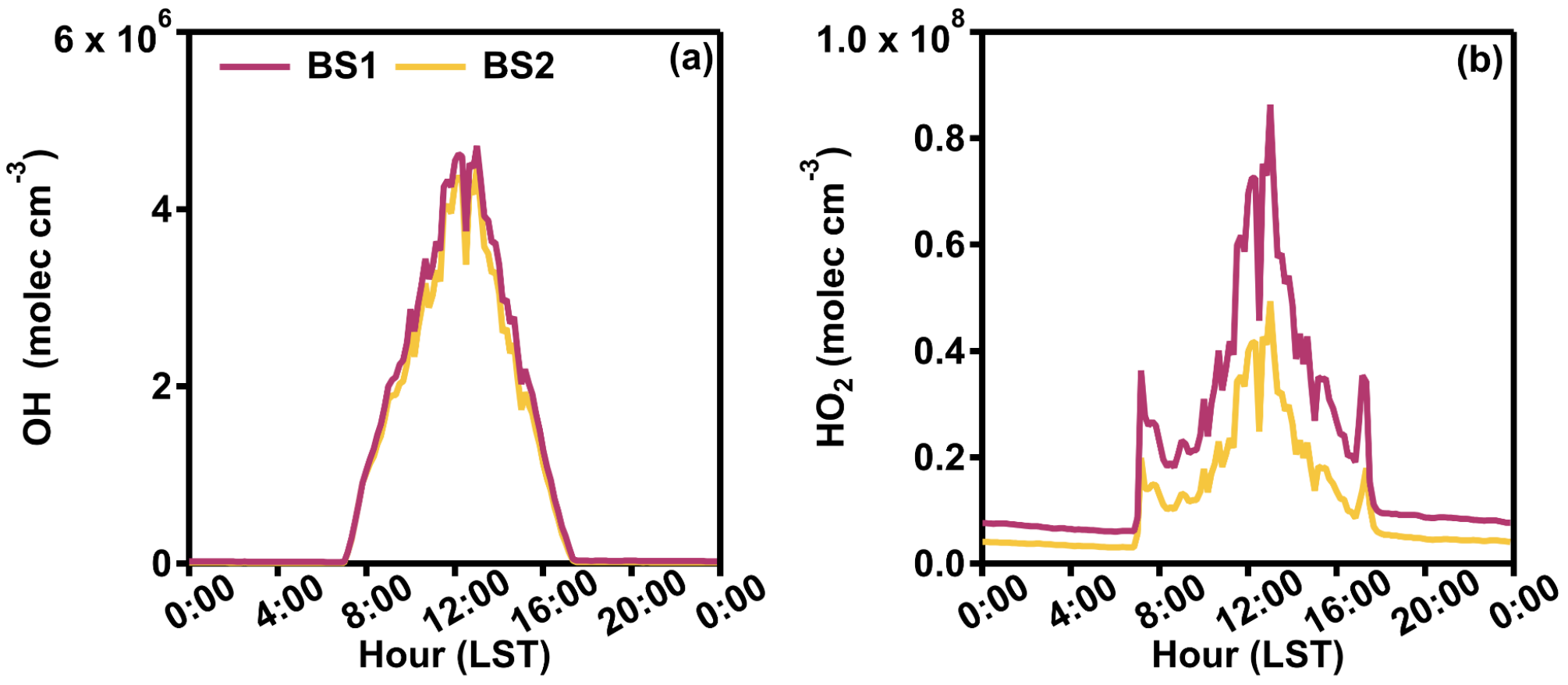

Figure S15. Diurnal profiles of simulated (a) OH and (b) HO_2 concentrations for BS1 (direct-PTR) and BS2 (GC-PTR).

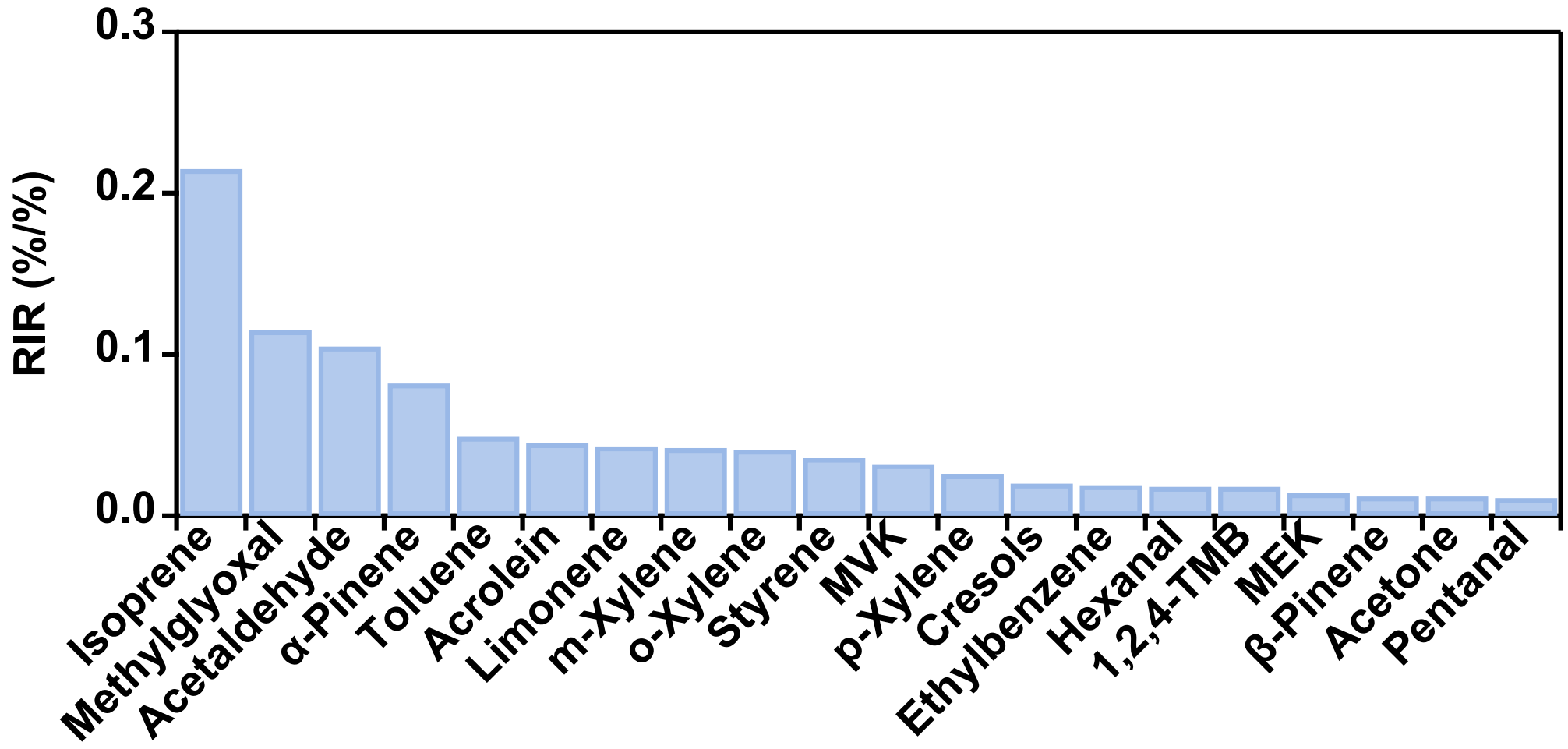

Figure S16.Top 20 RIR species determined by GC-PTR data set simulation in box model.

Table S1.Species identified by GC-PTR calibration and their RTs and RIs.

Compound	Formula	RIs	RTs (s)
Acetone	C_3H_6O	474	96.6
Isoprene	C_5H_8	520	69.9
Methyl Ethyl Ketone	C_4H_8O	577	182.2
Benzene	C_6H_6	640	213.2
m-Xylene	C_8H_{10}	855.4	367.0
1,2,4-Trimethylbenzene	C_9H_{12}	987	457.3
α-Pinene	$C_{10}H_{16}$	937	402.3
Methyl Vinyl Ketone	C_4H_6O	568	175.9

Table S2. Compounds identified in GC-PTR measurements.

Formula	Compounds	RT (s)
C_2H_4O	Acetaldehyde	9
C_3H_4O	Acrolein	86.6
C_3H_6O	Acetone	96.6
C_5H_8	Isoprene	69.9
C_6H_6	Benzene	213.2
C_7H_6O	Benzaldehyde	462.9
C_7H_8	Toluene	291.8
C_8H_8	Styrene	389
C_4H_6O	Methacrolein	153.9
	2-Butenal	228.8
	Methyl vinyl ketone	175.9
C_4H_8O	2-Methylpropanal	145.2
	Butanal	173.9
	2-Butanone	182.2
$C_5H_{10}O$	Pentanal	252.1
	Butanal, 3-methyl	189.9
	2-Pentanone	247.8
	3-Methyl-2-butanone	226.8
	3-Pentanone	253.1
$C_6H_{12}O$	Hexanal	321.4
	Pentanal, 2-methyl-	321.4
	Pentanal, 3-methyl-	303.4
	2-Hexanone	315.4
	Methyl Isobutyl Ketone	261.5
	3-Methyl-2-pentannoe	289.8
	2-Methyl-3-pentannoe	284.4
	3-Hexanone	294.8
$C_7H_{14}O$	Heptanal	400

	3-Hetanone	388.3
	2-Hetanone	394.7
C_8H_{10}	Ethylbenzene	361.4
	m/p-Xylene	367
	o-Xylene	387.7
$C_8H_{16}O$	Octanal	469.9
	3-Octanone	464
C_9H_{12}	1,2,3-Trimethylbenzene	479.6
	1,2,4-Trimethylbenzene	457.3
	2-Ethyltoluene	447.6
	4-Ethyltoluene & 135-Trimethylbenzene	436.3
	3-Ethyltoluene	431.3
$C_{10}H_{16}$	a-Pinene	402.3
	Camphene	418.7
	b-Pinene	438.6
	Carene	469.9
	Limonene	475.9
	y-Terpinene	481.3

Table S3. Summary of the sensitivities determined by direct-PTR calibration.

**Compound**	**Formula**	**Sensitivity (cps/ppb)**
Acetaldehyde	C_2H_4O	3211.0
Acrylonitrile	C_3H_3N	11718.5
Acrolein	C_3H_4O	8375.0
Acetone	C_3H_6O	13868.0
Methyl Vinyl Ketone	C_4H_6O	10587.0
Methyl Ethyl Ketone	C_4H_8O	10424.6
Isoprene	C_5H_8	2853.0
Methyl Isobutyl Ketone	$C_6H_{12}O$	4845.0
Benzene	C_6H_6	2920.9
Toluene	C_7H_8	7330.0
m-Xylene	C_8H_{10}	6960.0
Stryrene	C_8H_8	11000.6
1,2,4-Trimethylbenzene	C_9H_{12}	8003.2
α-Pinene	$C_{10}H_{16}$	2848.3

Table S4. Summary of the quantitative ions fraction (***f***), the estimated sensitivities, and the ions included in *f* calculations for non-calibrated species.

**Compounds**	***f***	**Calibration factors**	**Ions Included in *f* Calculation**
Methacrolein	0.92	10587	$C_4H_9O_2^+$
2-Butenal	0.93	11746.2	$C_4H_9O_2^+$
2-Methylpropanal	0.88	10926.7	$C_4H_{11}O_2^+$
Butanal	0.62	6414.4	$C_4H_{11}O_2^+$
2-Butanone	0.90	10435.0	$C_4H_{11}O_2^+$
3-Pentanone	0.88	9543.9	$C_5H_{11}O_2^+$
2-Pentanone	0.83	8642.0	$C_5H_{11}O_2^+$
3-Methyl-2-butanone	0.81	8314.9	$C_5H_{11}O_2^+$
Butanal, 3-methyl-	0.87	10261.6	$C_5H_{11}O_2^+$
Pentanal (GC Esti)	0.04	513.1	$C_5H_{11}O_2^+$; $C_5H_9^+$
2-Hexanone	0.85	11111.9	$C_6H_{13}O_2^+$
Hexanal	0.06	730.6	$C_6H_{13}O_2^+$; $C_6H_{11}^+$
Pentanal, 3-methyl-	0.22	2695.2	$C_6H_{11}^+$
Pentanal, 2-methyl-	0.65	7824.9	$C_6H_{13}O_2^+$; $C_6H_{11}^+$
2-Methyl-3-Pentanone	0.77	10086.2	$C_6H_{13}O_2^+$
3-Methyl-2-Pentanone	0.86	11235.6	$C_6H_{13}O_2^+$
3-Hexanone	0.90	11812.6	$C_6H_{13}O_2^+$
Heptanal	0.12	1407.0	$C_7H_{13}^+$; $C_7H_{17}O_2^+$; $C_5H_9^+$
2-Heptanone	0.66	8626.3	$C_7H_{17}O_2^+$
3-Heptanone	0.81	10574.2	$C_7H_{17}O_2^+$
3-Octanone	0.60	7999.9	$C_8H_{19}O_2^+$
Octanal	0.12	1435.1	$C_5H_9^+$; $C_8H_{15}^+$; $C_8H_{19}O_2^+$
o-Xylene	0.88	6469.7	$C_7H_7^+$
m&p-Xylene	0.85	7523.0	$C_7H_7^+$; $C_8H_9^+$
Ethylbenzene	0.34	2450.9	$C_6H_7^+$; $C_7H_7^+$
1,2,3-Trimethylbenzene	1.00	8195.0	—
2-Ethyltoluene	0.63	4771.8	$C_7H_9^+$
3-Ethyltoluene	0.62	4682.9	$C_7H_9^+$
4-Ethyltoluene&135-TMB	1.00	7539.4	—
Camphene	0.48	4090.9	$C_6H_9^+$
β-Pinene	0.44	3771.2	$C_6H_9^+$
Carene	0.39	3316.3	$C_6H_9^+$
γ-Terpinene	0.48	4058.5	$C_6H_9^+$
Limonene	0.63	5360.3	$C_6H_9^+$

Table S5. VOCs measured by PTR-ToF-MS and GC-MS/FID/ECD constrained in box model simulation.

Name	Formula	Data Source
Isoprene	C_5H_8	PTR-ToF-MS
Benzene	C_6H_6	PTR-ToF-MS
Toluene*	C_7H_8	PTR-ToF-MS
Styrene	C_8H_8	PTR-ToF-MS
o-xylene	C_8H_{10}	PTR-ToF-MS
m-xylene	C_8H_{10}	PTR-ToF-MS
p-xylene	C_8H_{10}	PTR-ToF-MS
Ethylbenzene	C_8H_{10}	PTR-ToF-MS
2-Ethyltoluene	C_9H_{12}	PTR-ToF-MS
3-Ethyltoluene	C_9H_{12}	PTR-ToF-MS
4-Ethyltoluene	C_9H_{12}	PTR-ToF-MS
1,2,3-Trimethylbenzene	C_9H_{12}	PTR-ToF-MS
1,2,4-Trimethylbenzene	C_9H_{12}	PTR-ToF-MS
1,3,5-Trimethylbenzene	C_9H_{12}	PTR-ToF-MS
a-Pinene	$C_{10}H_{16}$	PTR-ToF-MS
b-Pinene	$C_{10}H_{16}$	PTR-ToF-MS
Limonene	$C_{10}H_{16}$	PTR-ToF-MS
Acetaldehyde*	C_2H_4O	PTR-ToF-MS
Acrolein*	C_3H_4O	PTR-ToF-MS
Methylglyoxal*	$C_3H_4O_2$	PTR-ToF-MS
Acetone*	C_3H_6O	PTR-ToF-MS
Methyl vinyl ketone	C_4H_6O	PTR-ToF-MS
Methacrolein	C_4H_6O	PTR-ToF-MS
2-Butenal	C_4H_6O	PTR-ToF-MS
2-Butanone	C_4H_8O	PTR-ToF-MS
Butanal	C_4H_8O	PTR-ToF-MS
2-Methylpropanal	C_4H_8O	PTR-ToF-MS
Pentanal	$C_5H_{10}O$	PTR-ToF-MS
2-Pentanone	$C_5H_{10}O$	PTR-ToF-MS
3-Pentanone	$C_5H_{10}O$	PTR-ToF-MS
Phenol	C_6H_6O	PTR-ToF-MS
Hexanal	$C_6H_{12}O$	PTR-ToF-MS
2-Hexanone	$C_6H_{12}O$	PTR-ToF-MS
3-Hexanone	$C_6H_{12}O$	PTR-ToF-MS
Methyl Isobutyl Ketone	$C_6H_{12}O$	PTR-ToF-MS
Benzaldehyde*	C_7H_6O	PTR-ToF-MS
Cresols*	C_7H_6O	PTR-ToF-MS
Heptanal	$C_7H_{14}O$	PTR-ToF-MS
Ethane	C_2H_6	GC-MS/FID/ECD

Propane	C_3H_8	GC-MS/FID/ECD
i-Butane	C_4H_{10}	GC-MS/FID/ECD
n-Butane	C_4H_{10}	GC-MS/FID/ECD
i-Pentane	C_5H_{12}	GC-MS/FID/ECD
n-Pentane	C_5H_{12}	GC-MS/FID/ECD
2,2-Dimethylbutane	C_6H_{14}	GC-MS/FID/ECD
2,3-Dimethylbutane	C_6H_{14}	GC-MS/FID/ECD
2-Methylpentane	C_6H_{14}	GC-MS/FID/ECD
3-Methylpentane	C_6H_{14}	GC-MS/FID/ECD
n-Hexane	C_6H_{14}	GC-MS/FID/ECD
2-Methylhexane	C_7H_{16}	GC-MS/FID/ECD
3-Methylhexane	C_7H_{16}	GC-MS/FID/ECD
n-Heptane	C_7H_{16}	GC-MS/FID/ECD
n-Octane	C_8H_{18}	GC-MS/FID/ECD
n-Nonane	C_9H_{20}	GC-MS/FID/ECD
n-Decane	$C_{10}H_{22}$	GC-MS/FID/ECD
Cyclohexane	C_6H_{12}	GC-MS/FID/ECD
Ethene	C_2H_4	GC-MS/FID/ECD
Propene	C_3H_6	GC-MS/FID/ECD
i-Butene	C_4H_8	GC-MS/FID/ECD
1-Butene	C_4H_8	GC-MS/FID/ECD
1,3-butadiene	C_4H_6	GC-MS/FID/ECD
2-Methyl-1-Butene	C_5H_{10}	GC-MS/FID/ECD
3-Methyl-1-Butene	C_5H_{10}	GC-MS/FID/ECD
2-Methyl-2-Butene	C_5H_{10}	GC-MS/FID/ECD
1-Pentene	C_5H_{10}	GC-MS/FID/ECD
trans-2-Pentene	C_5H_{10}	GC-MS/FID/ECD
cis-2-Pentene	C_5H_{10}	GC-MS/FID/ECD
Ethyne	C_2H_2	GC-MS/FID/ECD

Reference

1. Pagonis, D.; Sekimoto, K.; de Gouw, J., A Library of Proton-Transfer Reactions of H3O+ Ions Used for Trace Gas Detection. *J. Am. Soc. Mass Spectrom.* **2019,** *30*, (7), 1330-1335.
2. Xue, L.; Wang, T.; Louie, P. K.; Luk, C. W.; Blake, D. R.; Xu, Z., Increasing external effects negate local efforts to control ozone air pollution: a case study of Hong Kong and implications for other Chinese cities. *Environ. Sci. Technol.* **2014,** *48*, (18), 10769-75.
3. Zeren, Y.; Guo, H.; Lyu, X.; Zhou, B.; Liu, X.; Yang, L.; Yuan, Z.; Wang, Y., Remarkable spring increase overwhelmed hard-earned autumn decrease in ozone pollution from 2005 to 2017 at a suburban site in Hong Kong, South China. *Sci. Total. Environ.* **2022**, 154788.
4. Wang, Y.; Wang, H.; Guo, H.; Lyu, X.; Cheng, H.; Ling, Z.; Louie, P. K. K.; Simpson, I. J.; Meinardi, S.; Blake, D. R., Long-term O_3–precursor relationships in Hong Kong: field observation and model simulation. *Atmos. Chem. Phys.* **2017,** *17*, (18), 10919-10935.
5. Lu, K.; Zhang, Y.; Su, H.; Brauers, T.; Chou, C. C.; Hofzumahaus, A.; Liu, S. C.; Kita, K.; Kondo, Y.; Shao, M.; Wahner, A.; Wang, J.; Wang, X.; Zhu, T., Oxidant (O_3 + NO_2) production processes and formation regimes in Beijing. *J. Geophys. Res.: Atmos.* **2010,** *115*, (D7).
6. Wang, W.; Yuan, B.; Su, H.; Cheng, Y.; Qi, J.; Wang, S.; Song, W.; Wang, X.; Xue, C.; Ma, C.; Bao, F.; Wang, H.; Lou, S.; Shao, M., A large role of missing volatile organic compound reactivity from anthropogenic emissions in ozone pollution regulation. *Atmos. Chem. Phys.* **2024,** *24*, (7), 4017-4027.
7. Feng, X.; Guo, J.; Wang, Z.; Gu, D.; Ho, K.-F.; Chen, Y.; Liao, K.; Cheung, V. T. F.; Louie, P. K. K.; Leung, K. K. M.; Yu, J. Z.; Fung, J. C. H.; Lau, A. K. H., Investigation of the multi-year trend of surface ozone and ozone-precursor relationship in Hong Kong. *Atmos. Environ.* **2023,** *315*, 120139.
8. Xue, L. K.; Wang, T.; Guo, H.; Blake, D. R.; Tang, J.; Zhang, X. C.; Saunders, S. M.; Wang, W. X., Sources and photochemistry of volatile organic compounds in the remote atmosphere of western China: results from the Mt. Waliguan Observatory. *Atmos. Chem. Phys.* **2013,** *13*, (17), 8551-8567.
9. Sarkar, C.; Sinha, V.; Kumar, V.; Rupakheti, M.; Panday, A.; Mahata, K. S.; Rupakheti, D.; Kathayat, B.; Lawrence, M. G., Overview of VOC emissions and chemistry from PTR-TOF-MS measurements during the SusKat-ABC campaign: high acetaldehyde, isoprene and isocyanic acid in wintertime air of the Kathmandu Valley. *Atmos. Chem. Phys.* **2016,** *16*, (6), 3979-4003.
10. Xue, L.; Gu, R.; Wang, T.; Wang, X.; Saunders, S.; Blake, D.; Louie, P. K. K.; Luk, C. W. Y.; Simpson, I.; Xu, Z.; Wang, Z.; Gao, Y.; Lee, S.; Mellouki, A.; Wang, W., Oxidative capacity and radical chemistry in the polluted atmosphere of Hong Kong and Pearl River Delta region: analysis of a severe photochemical smog episode. *Atmos. Chem. Phys.* **2016,** *16*, (15), 9891-9903.
11. Atkinson, R.; Arey, J., Atmospheric Degradation of Volatile Organic Compounds. *Chem. Rev* **2003,** *103*, (12), 4605-4638.
12. Koss, A. R.; Sekimoto, K.; Gilman, J. B.; Selimovic, V.; Coggon, M. M.; Zarzana, K. J.; Yuan, B.; Lerner, B. M.; Brown, S. S.; Jimenez, J. L.; Krechmer, J.; Roberts, J. M.; Warneke, C.; Yokelson, R. J.; de Gouw, J., Non-methane organic gas emissions from biomass burning: identification, quantification, and emission factors from PTR-ToF during the FIREX 2016 laboratory experiment. *Atmos. Chem. Phys.* **2018,** *18*, (5), 3299-3319.
13. Wu, C.; Wang, C.; Wang, S.; Wang, W.; Yuan, B.; Qi, J.; Wang, B.; Wang, H.; Wang, C.; Song, W.; Wang, X.; Hu, W.; Lou, S.; Ye, C.; Peng, Y.; Wang, Z.; Huangfu, Y.; Xie, Y.; Zhu, M.; Zheng, J.; Wang, X.; Jiang, B.; Zhang, Z.; Shao, M., Measurement report: Important contributions of oxygenated compounds to emissions and chemistry of volatile organic compounds in urban air. *Atmos. Chem. Phys.* **2020,** *20*, (23), 14769-14785.

14. Hui, L.; Feng, X.; Yuan, Q.; Chen, Y.; Xu, Y.; Zheng, P.; Lee, S.; Wang, Z., Abundant oxygenated volatile organic compounds and their contribution to photochemical pollution in subtropical Hong Kong. *Environ. Pollut.* **2023,** *335*, 122287.

15. Yuan, Q.; Zhang, Z.; Chen, Y.; Hui, L.; Wang, M.; Xia, M.; Zou, Z.; Wei, W.; Ho, K. F.; Wang, Z.; Lai, S.; Zhang, Y.; Wang, T.; Lee, S., Origin and transformation of volatile organic compounds at a regional background site in Hong Kong: Varied photochemical processes from different source regions. *Sci. Total. Environ.* **2024,** *908*, 168316.

16. Coggon, M. M.; Stockwell, C. E.; Claflin, M. S.; Pfannerstill, E. Y.; Xu, L.; Gilman, J. B.; Marcantonio, J.; Cao, C.; Bates, K.; Gkatzelis, G. I.; Lamplugh, A.; Katz, E. F.; Arata, C.; Apel, E. C.; Hornbrook, R. S.; Piel, F.; Majluf, F.; Blake, D. R.; Wisthaler, A.; Canagaratna, M.; Lerner, B. M.; Goldstein, A. H.; Mak, J. E.; Warneke, C., Identifying and correcting interferences to PTR-ToF-MS measurements of isoprene and other urban volatile organic compounds. *Atmos. Meas. Tech.* **2024,** *17*, (2), 801-825.
